# The Effect of Leflunomide on Cycling and Activation of T-Cells in HIV-1-Infected Participants

**DOI:** 10.1371/journal.pone.0011937

**Published:** 2010-08-03

**Authors:** Sarah W. Read, Mary DeGrezia, Emily J. Ciccone, Rebecca DerSimonian, Jeanette Higgins, Joseph W. Adelsberger, Judith M. Starling, Catherine Rehm, Irini Sereti

**Affiliations:** 1 Division of AIDS, National Institute of Allergy and Infectious Diseases, National Institutes of Health, Bethesda, Maryland, United States of America; 2 Laboratory of Immunoregulation, National Institute of Allergy and Infectious Diseases, National Institutes of Health, Bethesda, Maryland, United States of America; 3 Biostatistics Research Branch, Division of Clinical Research, National Institute of Allergy and Infectious Diseases, National Institutes of Health, Bethesda, Maryland, United States of America; 4 Science Applications International Corporation-Frederick, National Cancer Institute-Frederick, Frederick, Maryland, United States of America; 5 Department of Pharmacy, National Institutes of Health Clinical Center, National Institutes of Health, Bethesda, Maryland, United States of America; University of New South Wales, Australia

## Abstract

**Background:**

The pathogenesis of immunodeficiency due to human immunodeficiency virus (HIV)-1 is incompletely understood, but immune activation is believed to play a central role. Immunomodulatory agents that decrease immune activation may be useful in the treatment of HIV-1 infection.

**Methodology:**

A randomized, double blind, placebo-controlled pilot study of leflunomide for 28 days was performed in participants with HIV-1 infection who were not receiving antiretroviral therapy. Participants randomized to leflunomide were subsequently treated with cholestyramine until leflunomide levels were below detection limit.

**Findings:**

Treatment with leflunomide was well tolerated with mostly low-grade adverse events. Leflunomide administration reduced cycling of CD4 T cells (by ex vivo bromodeoxyuridine uptake and Ki67 expression) and decreased expression of activation markers (HLA-DR/CD38 co-expression) on CD8 T cells in peripheral blood. In addition, decreased expression of HIV-1 co-receptors was observed in both CD4 and CD8 T cells in the leflunomide group. There were no significant changes in naïve and memory T cell subsets, apoptosis of T cells or markers of microbial translocation.

**Conclusions:**

Leflunomide was effective in reducing immune activation in the setting of chronic HIV-1 infection suggesting that targeting immune activation with immunomodulatory agents may be a feasible strategy.

**Trial Registration:**

ClinicalTrials.gov NCT00101374

## Introduction

Infection with human immunodeficiency virus (HIV) leads to progressive loss of CD4 T cells and clinical immunodeficiency due to CD4 T cell lymphopenia. A number of observations suggest that CD4 T cell depletion in HIV is not only related to direct, virus-mediated death of infected cells but also to cell death of a large number of uninfected bystander T cells in the setting of increased activation that follows infection with HIV. The level of CD8 T cell activation can predict the rate of disease progression independent of HIV viral load [1], [2]. Furthermore, the level of immune activation prior to seroconversion has been shown to predict faster progression to AIDS [3].

Immune activation may be seen as a normal and necessary response to infection to most pathogens. However, in HIV infection, chronic immune activation and increased T cell turnover play a central role in the pathogenesis of AIDS. Both CD4 and CD8 T cell turnover rates are increased in HIV infected individuals [4], although HIV only infects and directly kills CD4 T cells, and CD8 T cell activation levels can predict the rate of disease progression independent of viral load [1], [2]. Simian immunodeficiency virus (SIV)-infected sooty mangabeys, the natural host of SIV, exhibit only minimally elevated levels of immune activation, and the majority do not develop CD4 T cell depletion or clinical disease despite high levels of virus replication [5]. In addition, patients receiving anti-retroviral therapy can maintain higher than normal levels of immune activation that may be associated with some of the chronic non-infectious complications of HIV [4], [6].

The cause of increased immune activation in HIV infection is an area of ongoing investigation and is likely multifactorial. Host immune responses to HIV and its antigens [7], direct effect of HIV proteins that bind to cellular proteins [8], translocation of microbial products across the intestinal mucosa[9], and increased levels of pro-inflammatory cytokines [10], [11], [12] are among the possible causes. Whatever the cause or causes, chronic generalized immune activation can have several detrimental consequences. It may serve to facilitate direct HIV infection of T cells, for example by promoting expression of CCR5 [13]. Likewise, chemokines and adhesion molecules are upregulated [14], leading to trafficking of T cells to areas where HIV replication is occurring and thereby providing more targets. Additionally, activation may lead to fibrosis in lymph nodes and lamina propria [15], [16], [17], interfering with reconstitution of those compartments. Inflammation and a state of hypercoaguability may also lead to other non-AIDS defining events such as myocardial infarction and stroke [18].

Taken together, these observations suggest that targeting HIV-associated immune activation may be a promising strategy in HIV infection. A number of immunomodulatory agents such as hydroxyurea [19], [20], mycophenolate mofetil [21], [22], and cyclosporine [23] have been studied with mixed results, with certain toxicities, risk of infection and lack of clear clinical efficacy preventing their use in clinical practice. Leflunomide is an immunomodulatory agent that is approved for the treatment of rheumatoid arthritis. The primary metabolite of leflunomide reversibly inhibits dihydroorotate dehydrogenase, the rate limiting step in the de novo synthesis of pyrimidines [24]. Unlike other cells, activated lymphocytes expand their pyrimidine pool by eight to sixteen-fold during proliferation and must use both salvage and de novo pathways of synthesis to meet this demand. In the presence of leflunomide, there is inhibition of T cell proliferation [25], [26]. By decreasing the supply of pyrimidines, leflunomide leads to interruption of cell cycle and decreased proliferation of activated lymphocytes and therefore is an interesting potential therapeutic agent in the treatment of HIV-1 infection.

Leflunomide has been studied in the treatment of other viral infections such as CMV and BK virus infections with promising results [27], [28]. In vitro studies have demonstrated an inhibitory effect of leflunomide on HIV replication in PBMCs alone and in combination with antiretroviral agents [29], [30], but to date there have been no reports of leflunomide treatment of HIV infected participants, whether directed at rheumatoid arthritis or viral infection.

In this study, we performed a randomized, double-blinded, placebo-controlled pilot study of leflunomide administered to HIV infected participants who were not on antiretroviral therapy in order to assess its safety and tolerability in this population as well as its effect on cycling and activation of CD4 and CD8 T cells.

## Methods

### Participants

HIV-1 infected persons ≥18 years of age were recruited from the NIAID outpatient HIV clinic. They were eligible to participate if they were not taking antiretroviral therapy (ART), had a CD4 T cell count ≥350 cells/mm^3^, a plasma HIV–RNA level ≥1000 copies/mL at the time of screening and a historical nadir CD4 T cell count of ≥200 cells/mm^3^. Routine laboratory results had to be within acceptable ranges. Specific exclusionary laboratory values were hemoglobin <10 g/dL, absolute neutrophil count <1000/mm^3^, platelets <100,000/mm^3^, and alanine aminotransferase, aspartate aminotransferase, or alkaline phosphatase >1.25 times the upper limit of normal. Because leflunomide can have significant hepatotoxicity participants were not receiving ART and were also excluded if they had liver disease, including chronic viral hepatitis, or were taking concomitant immunomodulatory agents or medications that could interact with leflunomide or cholestyramine. Pregnant or breast-feeding women were excluded, as were participants with a history of AIDS defining illness, neoplasm or acute bacterial infection. All participants provided written, informed consent. The protocol was approved by the NIAID Institutional Review Board, was monitored by a data safety monitoring board and was conducted under a United States FDA investigational new drug application (NCT00101374). The protocol, consent and IRB approval for this trial and supporting CONSORT checklist are available as supporting information; see Protocol S1, Consent S1, IRB Approval S1 and Checklist S1.

### Study design

The primary objective was evaluation of safety and secondary objectives included evaluation of biologic activity as measured by cycling and activation of T cells as well as expression of chemokine receptors. At entry, eligible participants were assigned by the central telephone (located in Pharmacy Department) using permuted block randomization in a 2∶1 ratio to receive leflunomide (Arava®, Sanofi Aventis) 20 mg orally (standard dose for RA) or matching placebo daily for 28 days. Loading with 100 mg orally for three days was not done to avoid acute side effects that could lead to unblinding and early withdrawal. The allocation sequence was concealed from all study staff. Participants and all study team members were blinded to treatment assignment during the 28-day treatment period. Treatment assignment was unblinded to the participant and clinic staff at the end of 28 days. Staff performing laboratory assays remained blinded until the completion of all assays. Because of the long half-life of leflunomide (2 weeks), those who were assigned to leflunomide underwent a washout procedure during which they were administered open-label cholestyramine (decreases leflunomide levels) for 11 days or longer until M1 (main leflunomide metabolite) levels were below the limit of detection to avoid potential synergistic hepatotoxicity if ART was started within the following weeks to months. The study safety and endpoint data were reviewed annually by an independent data and safety monitoring board. Participants were seen on days 1 (first day of study drug), 15 and 29 (end of study drug and unblinding) for clinical evaluation and study procedures. Leflunomide recipients were also seen on day 43 (after the wash out) and as needed if the first wash out was unsuccessful.

### Study evaluations

HIV-RNA levels were determined by ultrasensitive bDNA assay (Versant HIV-1 version 3.0, Siemens Corp., New York City). Leflunomide metabolite (A77 1726 or M1) levels were measured in plasma by Labcorp Clinical Trials Department (Burlington, NC) using high performance liquid chromatography. Immunophenotypic analysis was performed on days 1, 15 and 29 on whole blood by use of 4-color immunofluorescence as described elsewhere [31]. The following monoclonal antibodies were used for staining: CD3, CD4, CD8, CD27, CD45RO, CD38, HLA-DR, CCR5, and CXCR4 conjugated to fluorescein isothiocyanate, phycoerythrin, peridinin-chlorophyll-protein or allophycocyanin (Becton Dickinson (BD) Pharmingen, San Jose, CA). To study naive and memory phenotypes, subsets were defined by surface staining with CD3, CD4, CD45RO, and CD27 antibodies. Naive cells were defined as CD45RO^−^CD27^+^, central memory as CD45RO^+^CD27^+^, effector memory as CD45RO^+^CD27^−^, and effectors as CD45RO^−^CD27^−^. Annexin V and 7AAD staining was performed to assess for apoptosis as previously described [31]. Based on the mechanism of action of leflunomide, it was hypothesized that a decrease in effector or effector memory T cells could be seen. Staining for the nuclear antigen Ki67 was performed using Ki67 phycoerythrin (BD Immunocytometry Systems, San Jose, CA) following the protocol recommended by the manufacturer. Ex vivo bromodeoxyuridine (BrdU) staining was performed as previously described [32]. Plasma samples from days 1 and 29 were analyzed by ELFA (Enzyme Linked Fluorescent Assay) on a VIDAS instrument for D-Dimer (bioMerieux Inc., Durham, NC), by a single-plex kit (Meso Scale Discovery, Gaithersburg, MD) for C-reactive protein (CRP) and by real time PCR for *Cytomegalovirus* (CMV) DNA quantification as described by Yun et al [33]. Plasma levels of lipopolysaccharide (LPS) were also measured by the limulus assay as previously described [9] and soluble CD14 by commercially available ELISA assays (R&D Systems, Minneapolis, MN).

Samples were collected on an Epics XL (Beckman Coulter, Fullerton, CA), a FACSCanto (BD Immunocytometry Systems), or a FACSCalibur (BD Immunocytometry Systems) flow cytometer using CellQuest software (BD, San Jose, CA). Flow cytometry data were analyzed using FlowJo software (version 7.2.5, TreeStar Incorporated, Ashland, OR).

### Sample Size Considerations

The study was designed to compare the change from baseline to day 29 in the percentage of CD4 T cells expressing Ki67 between the two treatment groups with a 2∶1 randomization favoring leflunomide. For sample size calculation using previous data [4], the estimate of mean Ki67 expression was assumed to be 4.0% and the standard deviation of change to be 0.60. A sample size of fifteen would allow detection of a 30% drop in the mean Ki67 expression with 90% power using a two-sided t-test with type 1 error rate set to 0.05. To allow for 17% dropout, 18 participants were recruited.

### Statistical Analysis

Nonparametric tests were used for all analyses and median values are reported throughout. Changes from baseline within each group at 15 and 29 days were evaluated using the Sign test. Data comparisons between the two groups were performed using the Wilcoxon rank-sum test. Analyses were performed per protocol. Given the exploratory nature of this study, there was no adjustment for multiple testing.

## Results

### Participant characteristics

**Figure 1:**
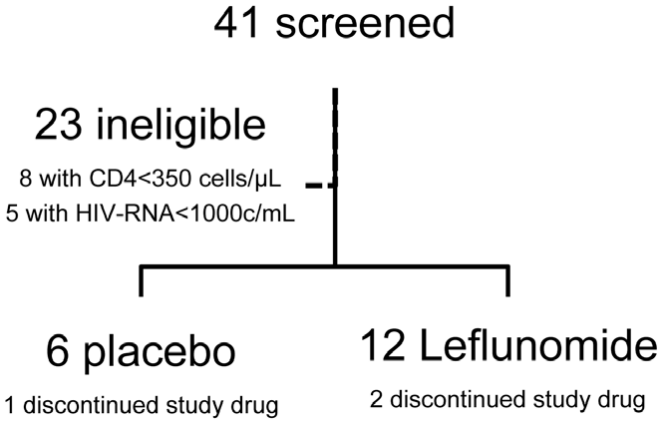
Flow chart of clinical study.

Forty-one potential participants were screened for the study between April 2005 and April 2007. Twenty-three participants were found ineligible. The majority had an exclusionary laboratory value, including eight participants with a CD4 T cell count less than 350 cells/mm^3^ and five participants with an HIV-RNA less than 1000 copies/mL. Eighteen participants were enrolled onto the study; 12 were assigned to leflunomide, and 6 were assigned to placebo (Figure 1). The two groups were well matched with no statistically significant differences in baseline characteristics (Table 1). Two participants randomized to the leflunomide arm discontinued treatment early. One participant developed oral ulcers on Day 2 that were judged to be probably unrelated to study treatment, and one participant chose to come off study early. Therefore there were 10 participants in the leflunomide group for per protocol analyses.

**Table 1:** Participant baseline characteristics^A^ (median value with interquartile range in parentheses).

	Leflunomide	Placebo
	(N = 10)	(N = 6)
Gender (%male)	90%	67%
Age (yrs)	40	39
	(26–44)	(30–43)
HIV-1 RNA (log_10_)	3.87	4.31
	(3.69–4.09)	(3.60–4.75)
%CD4	32	32
	(20–35)	(30–35)
CD4 T cells/µL	434	637
	(337–503)	(597–676)
%CD8	49	55
	(38–56)	(47–61)
CD8 T cells/µL	814	961
	(520–1063)	(932–1175)
4∶8 Ratio	0.70	0.58
	(0.37–0.92)	(0.46–0.70)

A There were no statistically significant differences in baseline characteristics between groups.

### Toxicity data

Two serious adverse events were reported during this study. One participant assigned to leflunomide experienced grade 3 neutropenia on Day 43. Because this participant had a history of neutropenia, had grade 2 neutropenia on a pre-entry visit, and developed this adverse event at a time when the leflunomide level was undetectable, this event was judged to be unrelated to treatment. A second participant, who was also assigned to leflunomide, was hospitalized on Day 54 for grade 3 depression and suicidal ideation. This participant had a history of depression and one previous suicidal gesture. The event was judged to be unrelated to treatment. There were no grade 4 signs, symptoms or laboratory abnormalities reported during this study.

Four participants in the leflunomide group reported three grade 2 events (headache, elevated lipase, and neutropenia) and nine grade 1 events (headache, fatigue, rash, dry mouth, taste alteration, abdominal cramping, increased amylase, increased lipase and hyperbilirubinemia) that were at least possibly related to leflunomide or placebo. Three of the six participants in the placebo group reported five grade 1 events (diarrhea, nausea, increased ALT, increased AST, and hyperbilirubinemia). In addition, two participants in the leflunomide group reported inability to comply with the cholestyramine washout as prescribed due to unpleasant taste and consistency and frequency of dosing.

### Leflunomide metabolite (A77 1726 or M1) levels

The Day 29 plasma M1 level achieved in this treatment group was 21.50 mg/L (range 12.3–41.0 mg/L). All participants in the leflunomide group were able to achieve levels below the limit of detection (<0.02 mg/L) following administration of cholestyramine, with the exception of two participants who were lost to follow up before a level of <0.02 mg/L could be documented. Five of the ten participants who completed 29 days of leflunomide required two courses of cholestyramine to achieve a level <0.02 mg/L.

### Changes in T cell counts and plasma HIV-RNA

**Figure 2:**
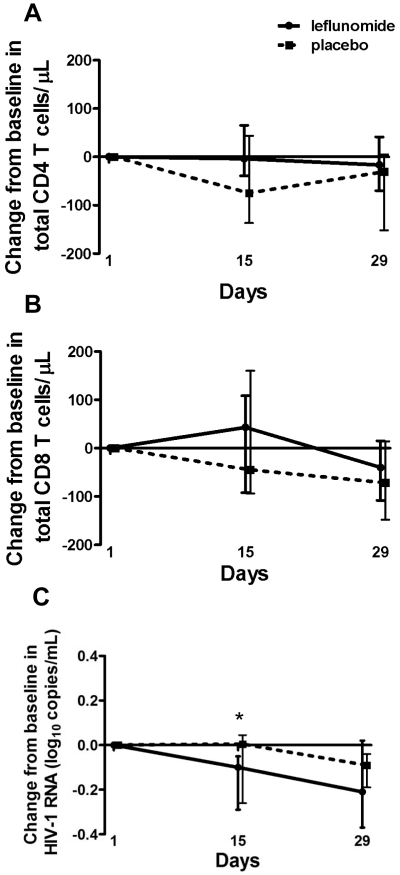
Changes in T cell counts and HIV viral load. A) Changes from baseline in CD4 T cell counts, and B) CD8 T cell counts throughout the study duration; median values and interquartile ranges; C) Change from baseline in HIV plasma viral load; median values and interquartile ranges. There was a significant decrease in HIV-1 RNA at Day 15 in the leflunomide group (*p-value<0.05).

There were no statistically significant changes in CD4 (Figure 2A) or CD8 (Figure 2B) T cell counts during the study. A statistically significant decrease in plasma HIV-RNA was seen in the leflunomide group at Day 15 (median change, −0.1 log_10_ copies/mL; p-value = 0.02), however the change from baseline in this group was not significant at Day 29 and was comparable to the change in the placebo group (Figure 2C).

### Changes in lymphocyte cycling and phenotype

**Figure 3:**
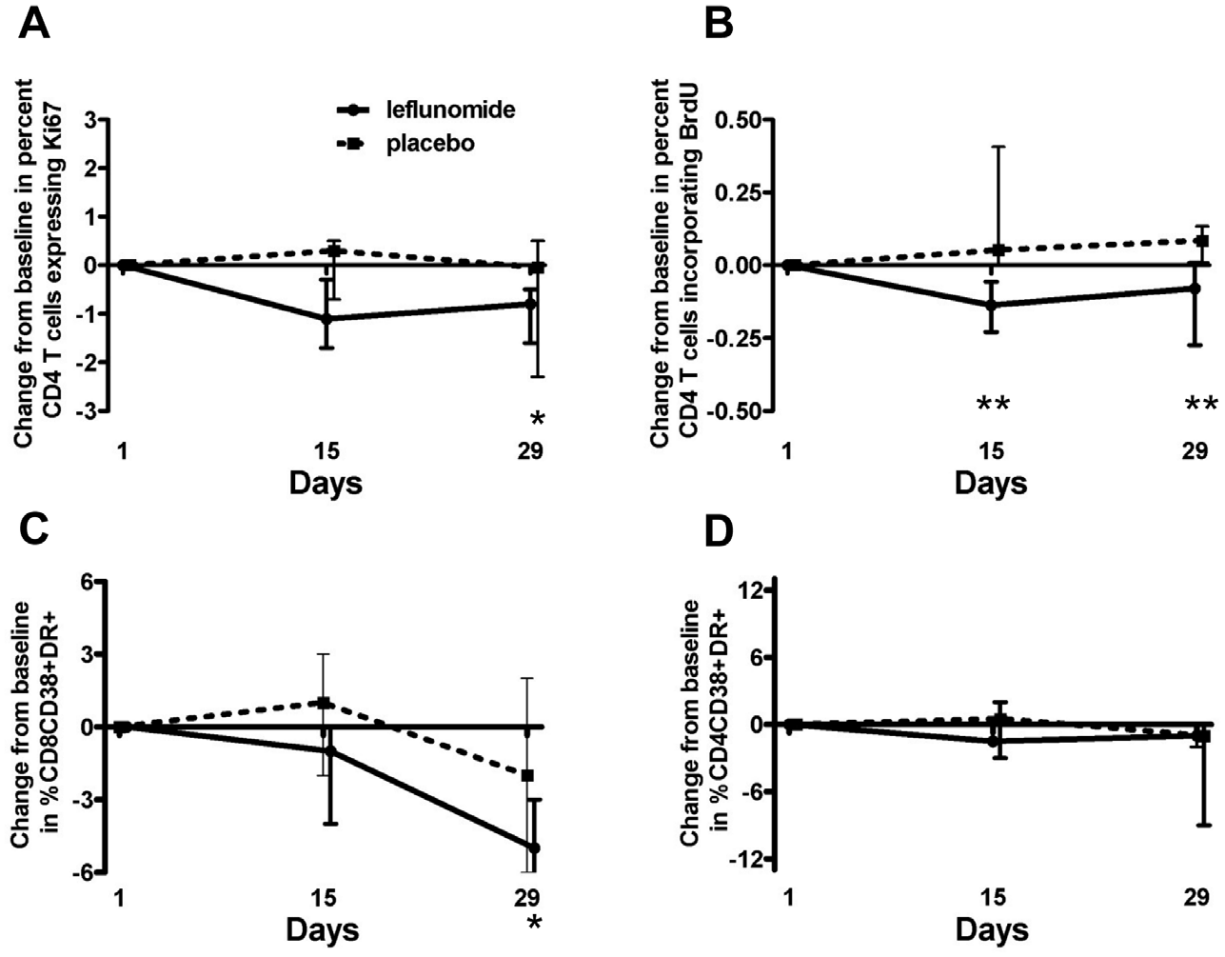
Changes in markers of CD4 T cell cycling and CD8 T cell activation. A) Change from baseline in percent Ki67 expression in CD4 T cells; median values and interquartile ranges. There was a significant decrease in Ki67 expression by CD4 T cells at Day 29 in the leflunomide group (*p-value<0.05). B) Change from baseline in percent BrdU incorporation by CD4 T cells; median values and interquartile ranges. There was a significantly greater change in BrdU incorporation by CD4 T cells in the leflunomide group compared to the placebo group at Day 15 and Day 29 (**p-value<0.05). C) Change from baseline in percent of activated CD8 T cells (%CD8CD38+HLADR+), median values and interquartile ranges. There was a significant decrease in activated CD8 T cells at Day 29 in the leflunomide group (*p-value<0.05). D) Change from baseline in percent of CD4 T cells co-expressing CD38 and HLA-DR, median values and interquartile ranges.

Expression of Ki67, a nuclear antigen expressed by cells in cell cycle, was also comparable between the two groups at baseline (leflunomide 4.3% vs placebo 5.6%, p-value = 0.79) (Table 2). Ki67 expression decreased significantly in the leflunomide group from baseline to Day 29 (median change, −0.8%; p-value = 0.02) (Figure 3A) and remained stable in the placebo group (median change, −0.05%; p-value = 1.0). However, the comparison of changes in Ki67 expression in CD4 T cells between the two treatment groups did not reach statistical significance (p-value = 0.55).

**Table 2:** Participant baseline levels of turnover, activation and coreceptor expression^A^ (median value with interquartile range in parentheses).

	Leflunomide	Placebo
%CD4+Ki67+	4.3 (3.9–7.2)	5.6 (4.1–7.0)
%CD4+BrdU+	0.37 (0.22–0.43)	0.26 (0.12–0.55)
%CD4+CD38+DR+	9 (6–13)	11 (8–26)
%CD4+CCR5+	14 (9–19)	16 (9–22)
%CD4+CXCR4+	90 (88–91)	77 (63–89)
%CD8+Ki67+	3.0 (2.3–3.6)	4.7 (1.4–7.3)
%CD8+BrdU+	0.18 (0.13–0.25)	0.31 (0.09–0.54)
%CD8+CD38+DR+	39 (35–53)	46 (31–62)
%CD8+CCR5+	41 (32–51)	40 (29–61)
%CD8+CXCR4+	74 (62–83)	60 (40–65)

A There were no statistically significant differences in baseline immunologic variables between groups.

Ex-vivo BrdU incorporation, another marker of cell cycling (in S phase), was also examined. Baseline values for CD4 T cells did not differ between groups (Table 2). BrdU incorporation in the CD4 T cells in the leflunomide group decreased significantly at Day 15 and Day 29 from baseline (change at Day 15, −0.14% and at Day 29, −0.08%). This decrease was significantly different (p = 0.03) from the changes seen in the placebo group (at Day 15, 0.05% and at Day 29, 0.09%) (Figure 3B).

There were no differences between groups in frequency of activated CD8 T cells, co-expressing markers of activation CD38 and HLA-DR, at baseline (Table 2). A decrease in frequency of activated CD8 T cells was observed in the leflunomide group at Day 29 (median change, −5%; p-value = 0.02) (Figure 3C) but not in the placebo group. Activated CD4 T cells (HLA-DR+CD38+) did not decrease in either group (Figure 3D).

**Figure 4:**
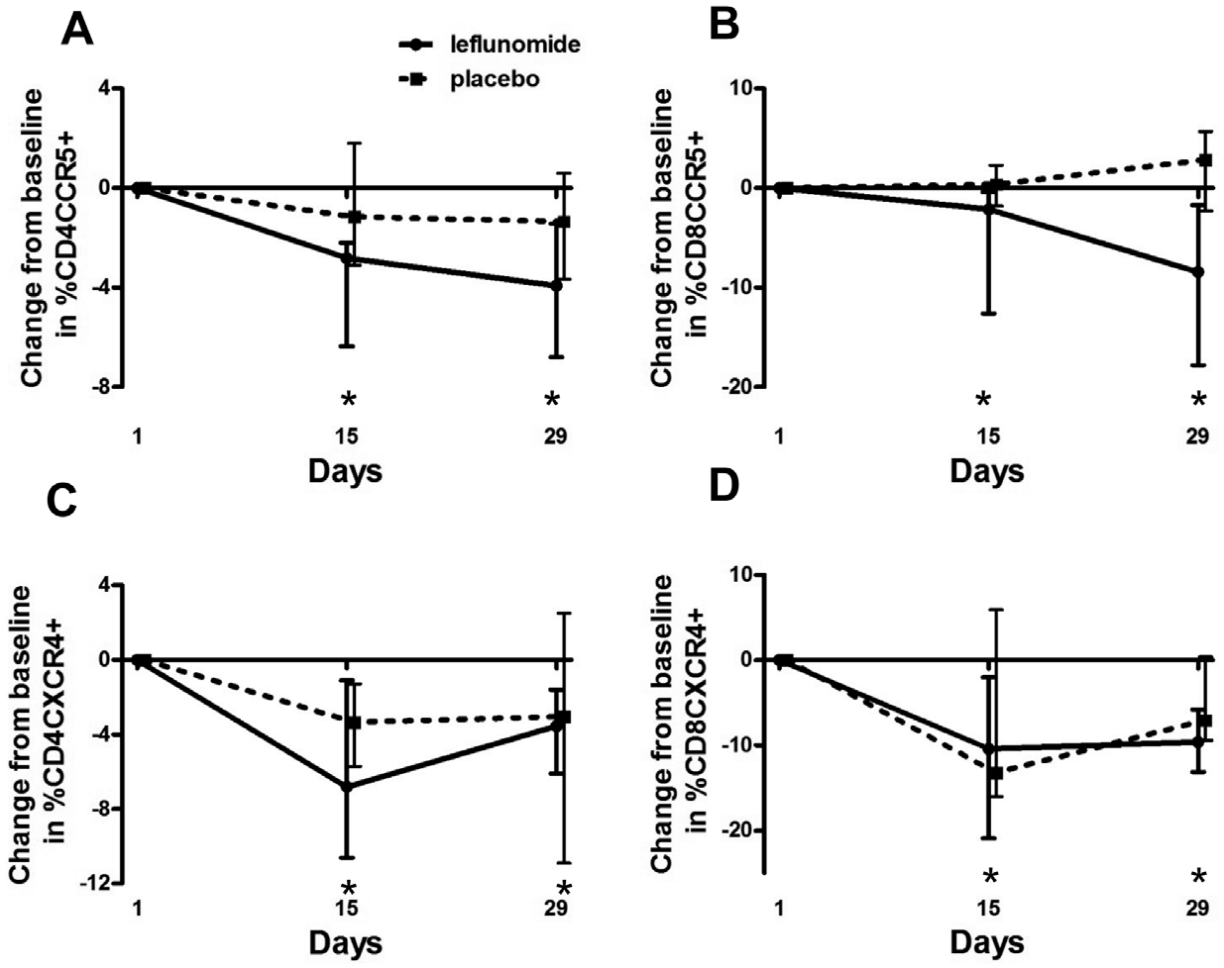
Changes in HIV coreceptor expression. A) Change from baseline in percent of CD4 T cells, and B) CD8 T cells expressing CCR5, median values and interquartile ranges. In the leflunomide group, there were significant decreases in CCR5 expression by CD4 and CD8 T cells at Day 15 and Day 29 (*p-value<0.05). C) Change from baseline in percent of CD4 and D) CD8 T cells expressing CXCR4; median values and interquartile ranges. In the leflunomide group, there were significant decreases in CXCR4 expression by CD4 and CD8 T cells at Day 15 and Day 29 (*p-value<0.05).

Expression of the HIV-1 co-receptors CCR5 and CXCR4 by CD4 and CD8 T cells did not differ significantly at baseline (Table 2). In the leflunomide group, there were significant decreases in frequency of both CD4 (Figure 4A) and CD8 (Figure 4B) T cells expressing CCR5 at Day 15 (CD4: −2.8%, p-value = 0.02; CD8: −2.2%, p-value = 0.02) and Day 29 (CD4: −3.9%, p-value = 0.02; CD8: −8.5%, p-value = 0.02) as compared to baseline, whereas no significant changes were seen in the placebo group at Day 15 or Day 29. Decreases in expression of CXCR4 were also seen in both CD4 (Figure 4C) and CD8 (Figure 4D) T cells in the leflunomide group at Day 15 (CD4: −6.8%, p-value = 0.02; CD8: −10.4%, p-value = 0.02) and Day 29 (CD4: −3.6%, p-value = 0.02; CD8: −9.6%, p-value = 0.002) but not in the placebo group at either time point.

Proportions of CD4 or CD8 T cells with naïve, central memory, effector memory, or effector phenotype did not change significantly in either group (data not shown). There were also no changes seen in either group in frequency of apoptotic cells as measured by Annexin V or 7AAD staining, plasma LPS, soluble CD14, D-dimer and CRP levels (data not shown). Plasma CMV PCR was positive in a very small number of participants (N = 6) at barely detectable levels to allow any meaningful comparisons.

## Discussion

In this study, short-term administration of leflunomide to HIV-1 infected participants not receiving antiretroviral therapy was overall well tolerated and led to decreases in CD4 T cell cycling and CD8 T cell activation as well as decreases in expression of both CCR5 and CXCR4 without any significant effects on T cell counts or plasma HIV-1 levels.

Despite the short exposure and the overall low plasma levels of leflunomide a decrease in cycling of CD4 T cells was observed in the treatment group. A decrease in both BrdU incorporation by CD4 T cells and CD38/HLA-DR expression on CD8 T cells was also seen in the treatment group compared to the placebo group indicating a decrease in T cell cycling and activation. Rates of apoptosis were not affected, nor were markers of microbial translocation (LPS, sCD14) or inflammation (CRP), supporting the role of leflunomide in inhibiting cycling of activated lymphocytes as the main mechanism behind the decrease in activation levels. Similar to results from in vitro studies published previously [30], expression of HIV-1 co-receptors CCR5 and CXCR4 also decreased following leflunomide treatment. A decrease in CCR5 expression on CD4 T lymphocytes may be a desirable outcome in better controlling chronic HIV-associated activation as suggested by the non-pathogenic SIV primate models [34].

The overall low levels achieved at Day 29 (21.5 mg/L) compared to the steady-state levels expected in treatment of rheumatoid arthritis (50–100 mg/L) may have blunted T cell effects. Studies of the treatment of both rheumatoid arthritis and BK virus infection have demonstrated a concentration-effect relationship with leflunomide [35], [36]. Although there is marked inter-individual variability in the pharmacokinetics of leflunomide, it is likely that a longer course of therapy in this study could have resulted in higher levels and potentially a more dramatic effect on T cell activation. Unfortunately, clinical use of leflunomide is not without risks and the prolonged half-life makes its use very complicated. There is potential for hepatotoxicity and pancytopenia as well as potential teratogenicity, making it an unattractive drug to combine with ART. In this study though, in a very rigorously selected group of HIV+ participants, a short course was safe and well-tolerated in the absence of ART, but we doubt its potential for broader use and applicability.

In conclusion, leflunomide was well tolerated and effective in decreasing T cell turnover in HIV-infected participants but potential toxicities and long half-life would limit further study as possible adjunct immune therapy in HIV. However newer drugs with similar mechanisms of action could potentially be further pursued as an experimental treatment to decrease immune activation in HIV infection either as adjunct therapy to ART to decrease immune activation in treated patients or potentially to defer ART initiation.

## Supporting Information

Protocol S1 Trial protocol.
(0.37 MB DOC)

IRB Approval S1 (0.06 MB PDF)

CROI Abstract S1 (0.06 MB PDF)

Consent S1 (0.12 MB DOC)

Checklist S1 CONSORT Checklist.
(0.19 MB DOC)
