# Supplementary material for: The Effect of Leflunomide on Cycling and Activation of T-Cells in HIV-1-Infected Participants
Source: PLoS One. 2010 Aug 3;5(8):e11937. doi: 10.1371/journal.pone.0011937 (PMC2914784; doi:10.1371/journal.pone.0011937)
Supplement: Protocol S1 — Trial protocol. (0.37 MB DOC) [file pone.0011937.s001.doc]

A Double-Blind, Randomized, Placebo-Controlled Study to Evaluate the Effect of Leflunomide on HIV-1 Associated Immune Proliferation In Vivo

**NIAID Protocol Number: 05-I-0065**

**IND Number: RCHSPB, NIAID, #71,183**

**Version: 6.0**

**Date: 07/21/2006**

**Abbreviated Title: Leflunomide and Tcell turnover**

**Key words: Immunomodulatory Drug, Antiviral, Ki67+,**

**Immune Activation**

# Principal Investigator: Irini Sereti, M.D., MHs

# NIAID, LIR

Building 10, Room 11B04

Bethesda, MD 20892

Phone: 301-496-5533

Email: [isereti@niaid.nih.gov](mailto:isereti@niaid.nih.gov)

**Accountable investigator: Richard Davey, Jr., M.D.**

NIAID, LIR

Building 10, Room 11C103

Bethesda, MD 20892

Phone: 301-496-8029

Email: [rdavey@niaid.nih.gov](mailto:rdavey@niaid.nih.gov)

**Z number: AI000865**

# Associate Investigators: Sarah Read, M.D.

# NIAID, LIR

Building 10, Room 11B05

Bethesda, MD 20892

Phone: 301-451-2402

# Email: [**readsa@niaid.nih.gov**](mailto:swynne@niaid.nih.gov)

**H. Cliff Lane, M.D.**

NIAID, OCR

Building 10, Room 11S231

Bethesda, MD 20892

Phone: 301-496-7196

Email: [clane@niaid.nih.gov](mailto:clane@niaid.nih.gov)

**Alice Pau, PharmD**

NIAID, OCR

Building 10, Room 11C103

Bethesda, MD 20892

Phone: 301-451-3740

Email: [apau@niaid.nih.gov](mailto:apau@niaid.nih.gov)

**Joseph Kovacs, M.D.**

NIAID, CC/CCMD

Building 10, Room 4D04

Bethesda, MD 20892

Phone: 301-496-9907

Email: [jkovacs@niaid.nih.gov](mailto:jkovacs@niaid.nih.gov)

**Protocol Statistician: Dean Follman, PhD**

NIAID, BRB/OCR

6700 A Rockledge Drive, Room 5138

Bethesda, MD 20892

Phone: 301-451-5128

Email: dfollman@niaid.nih.gov

**Study Coordinator: Wendy Gao, R.N., M.S.**

NIAID

Building 10, Room 8C418

Bethesda, MD 20892

Phone: 301-435-

Email: mdegrezia@niaid.nih.gov

**Persons authorized to**

**obtain consent: PI, AIs**

**JoAnn Mican, OCR, NIAID**

**Michael Polis, LIR, NIAID**

**Henry Masur, CCMD, CC**

**Mary Wright, OCR, NIAID**

**Frank Maldarelli, NCI**

**Stephen Migueles, LIR, NIAID**

**Richard Davey, LIR, NIAID**

**Jorge Tavel, LIR, NIAID**

**Michael Sneller, LIR, NIAID**

**Mark Parta, SAIC**

**Research facility where the**

**investigation will be conducted: National Institutes of Health, Clinical Center**

National Institute of Allergy and Infectious

Diseases- HIV Clinic

Building 10, 8th floor clinic (OP-8)

10 Center Drive

Bethesda, MD 20892

# Clinical laboratory facilities

**to be used in the investigation: Department of Laboratory Medicine**

Dr Thomas Fleisher, Chief

National Institutes of Health

Building 10- Room 2C306

Bethesda, MD 20892

NCI- Frederick Cancer Research & Development Center

Dr Michael Baseler- Head Clinical Services Program

Fort Detrick

Boyles Street, Building 1050

Frederick, MD 21702-1201

**Labcorp Clinical Trials Department**

1904 Alexander Drive

RTP, NC 27709

# Sponsor: Regulatory Compliance and Human Subjects

**Protection Branch (RCHSPB)**

NIAID, OCR, OD

6700-B Rockledge Drive, MSC 7609

Bethesda, MD 20892

**Precis**

Increased T cell turnover is one of the main abnormalities observed in HIV infected patients and one of the main mechanisms leading to CD4 lymphopenia. This has led to the hypothesis that medications that act directly to suppress immune activation and normalize T cell turnover, could be used in HIV infection. The purpose of this protocol is to evaluate the effect of the immunomodulatory agent, leflunomide, on CD4+ T cell proliferation in HIV infected adults. HIV infected adults who have stable HIV viral loads and are not taking antiretrovirals will receive leflunomide or placebo for 28 days. CD4+ T cell proliferation will be measured as percent Ki67 expression, and the change in expression from baseline to day 28 will be compared between groups. Various studies measuring immune parameters such as CD4+ and CD8+ T cells counts and level of activation will be collected as well as safety studies and HIV viral loads. The primary study risk is adverse reaction to leflunomide. The study will be double-blinded randomized 2:1 (leflunomide versus placebo) and will be reviewed by a DSMB. Total enrollment for the study will be 18 patients.

Table of Contents

1.0 Background 7

1.1 CD4+ T cell depletion in HIV infection 7

1.2 Immunomodulatory agents in the treatment of HIV infection 7

1.3 De novo pyrimidine synthesis 9

1.4 Leflunomide: metabolism, mechanism of action, and use in rheumatoid arthritis 9

1.5 Leflunomide and HIV *in vitro* 10

1.6 Leflunomide and potential clinical use 11

2.0 Hypothesis 11

3.0 Study Objectives 11

3.1 Primary objective 11

3.2 Secondary objectives 11

4.0 Study Design and Procedures 12

4.1 Design 12

4.2 Endpoints 12

4.2.1 Primary endpoint 12

4.2.2 Secondary endpoints 12

4.3 Sample Size and analysis 12

4.4 Procedures 13

4.4.1 Screening period 13

4.4.2 Treatment period (Day 1 through 28) 14

4.4.3 Post treatment follow up period (Day 29 through 64) 15

5.0 Study Population [17](#__RefHeading___Toc484061104)

5.1 Inclusion Criteria [17](#__RefHeading___Toc484061105)

5.2 Exclusion Criteria [18](#__RefHeading___Toc484061106)

6.0 Patient Monitoring and Criteria for Withdrawal from Study [19](#__RefHeading___Toc484061107)

6.1 Patient monitoring 19

6.2 A77 1726 measurement 20

6.3 Criteria for stopping study drug and immediate institution of drug elimination procedure 20

6.4 Criteria for stopping enrollment 22

7.0 Adverse Events Reporting 22

7.1 Definitions 22

7.2 Reporting 23

7.2.1 Reporting to the IRB 23

7.2.2 Reporting to the sponsor 24

8.0 Protocol Monitoring 25

8.1 Data Safety Monitoring Board 25

9.0 Human Subjects Protection 25

9.1 Subject selection criteria 25

9.2 Benefits 26

9.3 Risks 26

9.3.1 Specific medication risk 26

9.3.1.1 Leflunomide 26

9.3.1.2 Cholestyramine 29

9.3.2 Other risks 30

9.4 Compensation [31](#__RefHeading___Toc484061111)

9.5 Consent/Assent Procedures 31

9.6 Confidentiality 31

10.0 References 31

11.0 Appendices 35

11.1 Appendix A - Medications that are metabolized by CYP 2C9 and contraindicated during treatment period 35

11.2 Appendix B - Medications that have interactions with cholestyramine 36

11.3 Appendix C - CDC 1993 Revised classification system for HIV infection and expanded surveillance case definition for AIDS among adolescents and adults 38

11.4 Appendix D AIDS Clinical Trials Group (ACTG) toxicity table..........................40

**1.0 Background**

## **1.1 CD4+ T cell depletion in HIV infection**

Infection with HIV leads to progressive destruction of CD4+ T cells and clinical immunodeficiency due to CD4+ T cell lymphopenia. There is also a state of increased immune activation that leads to accelerated turnover and death of CD4+ T cells (1,2). Following the initiation of HAART, the rate of turnover decreases but remains abnormally elevated compared to healthy adults despite viral load levels of <50 copies/mL. The direct HIV-mediated killing of CD4+ T cells cannot account for the CD4 lymphopenia observed in AIDS or the inability to restore CD4 counts after viral load suppression to <50 copies/ml. A number of observations have been made that suggest that CD4+ T cell depletion in AIDS is not only related to direct, virus mediated death of infected cells but also to cell death of large numbers of uninfected bystander T cells in the setting of increased activation that follows infection with HIV (1,3).

Several observations have been made that support the theory that chronic immune activation and increased T cell turnover play a central role in the pathogenesis of AIDS. Both CD4+ and CD8+ T cell turnover rates are increased in HIV infected individuals although HIV only infect and directly kills CD4+ T cells (4). Furthermore, CD8+ T cell activation levels can predict the rate of disease progression independent of HIV viral load (5). Level of immune activation prior to seroconversion has also been described to predict faster progression to AIDS (6). Furthermore, the evidence correlating HIV viral load to rate of CD4+ T cell depletion can also be explained if levels of HIV replication correlate with increased level of immune activation and T cell turnover (7-9). Consistent with the idea that HIV-associated CD4+ T cell depletion is not simply the direct result of virus-mediated killing is that SIV-infected sooty mangabeys exhibit only minimally elevated levels of immune activation and do not develop CD4+ T cell depletion despite high levels of virus replication (10).

These observations support the use of immunomodulatory agents in the treatment of HIV infection. Agents such as hydroxyurea and mycophenolate mofetil have been investigated as potential therapeutic agents for HIV infection, however toxicities, risk of infection and lack of clear clinical efficacy have prevented their use in clinical practice.

## **1.2 Immunomodulatory agents in the treatment of HIV infection**

When resting latently infected CD4+ T cells become activated and proliferate, the HIV virus is able to replicate through incorporation with the host cell genome. Therefore, reduction of the number of activated T cells through use of immunomodulatory agents may decrease overall production of the virus. Among the agents that have been evaluated as possible immunomodulating treatment for HIV infection are hydroxyurea, mycophenolate mofetil, steroids, cyclosporine and rapamycin.

Hydroxyurea acts by inhibiting the enzyme ribonucleotide reductase. This inhibition leads to depletion of intracellular deoxynucleotide triphosphates that are necessary for cell division as well as HIV replication. Hydroxyurea leads to cell cycle arrest in G1/S phase and decreases cellular activation. *In vitro* studies on HIV infected lymphocytes have shown that hydroxyurea inhibits viral DNA synthesis (11) and acts synergistically with nucleoside reverse transcriptase inhibitors (12). Clinical studies with hydroxyurea have generally been in combination with didanosine based on the fact that hydroxyurea leads to an increase in the amount of didanosine metabolite and enhanced incorporation into the elongating DNA strand. Though early clinical studies demonstrated that hydroxyurea monotherapy was not effective in decreasing plasma HIV RNA, in combination with didanosine significant reductions in viral load were observed (13,14). Despite these findings, toxicities such as pancreatitis, lactic acidosis and bone marrow suppression have made hydroxyurea an unattractive therapeutic option.

Mycophenolate mofetil inhibits the de novo synthesis of deoxyguanosine triphosphate. Its effects are selective for lymphocytes that depend on de novo synthesis of purines and cannot use salvage pathways for guanosine synthesis. Mycophenolate potentiates the antiviral activity of guanosine inhibitors of reverse transcriptase such as abacavir by depleting the substrate for reverse transcriptase, thus inhibiting HIV replication (15). In addition it induces apoptosis of activated CD4+ T cells, another mechanism by which it may inhibit HIV replication.

Mycophenolic acid has been tested in combination with nucleoside reverse transcriptase inhibitors such as abacavir and didanosine in small pilot studies of patients with multidrug resistant HIV infection (16,17). CD4+ and CD8+ T cell counts remained stable with no change or decrease in plasma HIV RNA. The role of mycophenolate in the treatment of HIV remains unclear and is being studied further.

Corticosteroids have also been studied in HIV infected patients. One uncontrolled study published in 1995 indicated that prolonged administration of corticosteroids resulted in sustained increases in CD4 counts (18). The authors recently published a follow up article in which they retrospectively compared these patients who received prednisolone to a control group matched for baseline CD4+ T cell count and viral load (19). They showed that the prednisolone treated patients had an increase in CD4 + T cell counts of over 200 cells/mm^3^ in the first two weeks of treatment. On average the counts remained elevated for at least two years. The prednisolone treated patients also showed decreased levels of markers of apoptosis and cell activation as measured by CD25 and HLA-DR expression.

Given the numerous adverse effects of corticosteroids, particularly the increased incidence of avascular necrosis (AVN) of the hip in HIV-infected patients (20), there is heightened concern over the safety of corticosteroid use in the treatment of HIV. A multicenter study of the effects of prednisone on CD4+ T cell count and HIV viral load (ACTG 349) was closed prematurely when information was released about the increased incidence of hip AVN in association with steroids in HIV infected patients (21). In the 24 patients who had been randomized and completed at least 8 weeks therapy, those treated with prednisone experienced a median increase of 45% over baseline in CD4+ T cell count compared to control subjects who had only a 1% increase. The difference, however, did not reach statistical significance. There were also no statistically significant differences in markers of cell activation (HLA-DR and CD38). Furthermore, of 11 patients who volunteered to undergo MRI, two were found to have avascular necrosis. Given these findings, it is not clear what role if any glucocorticoids will play in the treatment of HIV.

## **1.3 De novo pyrimidine synthesis**

Unlike other cells, activated lymphocytes expand their pyrimidine pool by eight to sixteen-fold during proliferation and must use both salvage and de novo pathways of synthesis to meet this demand (22). Pyrimidine synthesis begins with the conversion of glutamine and ATP. Carbamoyl phosphate then condenses with aspartate to form N-carbamoylaspartate which is then converted to the pyrimidine ring dihydroorotate. Dihydroorotate diffuses into the mitochondria where the enzyme dihydroorotate dehydrogenase is located. This enzyme uses quinone as a cofactor to produce orotate which diffuses out of the mitochondria. Orotate is converted into uridine monophosphate (rUMP) in the cytoplasm. Inhibition of de novo rUMP synthesis at the level of dihydroorotate dehydrogenase would lead to decreased levels of rUMP and cells would arrest in the G1 phase of the cell cycle. The metabolite of the immunomodulatory agent leflunomide acts by inhibiting dihydrooratate dehydrogenase and has been shown to cause this effect on activated lymphocytes.

**1.4 Leflunomide: metabolism, mechanism of action, and use in rheumatoid arthritis**

Leflunomide is an isoxazole immunomodulatory agent that was approved by the FDA in 1998 for the treatment of rheumatoid arthritis. Following oral administration, leflunomide is rapidly and almost completely converted to an open-ring metabolite A77 1726, also referred to as M1 (23). The specific site of metabolism is unknown but in vivo and in vitro studies suggest a role for both the GI wall and the liver in drug metabolism. No specific enzyme has been identified as the primary route of metabolism for leflunomide; however, hepatic cytosolic and microsomal cellular fractions have been identified as sites of drug metabolism. M1 has also been identified as an inhibitor of cytochrome P450 isoenzyme 2C9. The primary metabolite, M1, is eliminated by renal excretion as well as by direct biliary excretion and has a half-life of approximately 2 weeks. Because of the long half-life, a drug elimination procedure (cholestyramine 8 g three times daily for 11 days) is recommended to expedite the removal of drug from plasma in the case of unexpected pregnancy or adverse event while receiving leflunomide. Administration of cholestyramine reduces the half-life of leflunomide to one day, most likely by interfering with biliary recycling of M1.

The M1 metabolite is a malononitrilamide to which most of the immunomodulatory effects of leflunomide are attributed. At low therapeutically applicable doses, M1 reversibly inhibits dihydroorotate dehydrogenase (DHODH), the rate limiting step in the de novo synthesis of pyrimidines. This results in inhibition of orotate production and decreased de novo synthesis of rUMP. The diminished level of rUMP results in p53 activation and translocation into the nucleus. After activation of p53, the damaged T cell will arrest in late G_1_ and not enter the S phase of the cell cycle, required for cell replication (24). As a result, there is inhibition of T cell proliferation resulting in decreased immune response.

The mechanisms of antiproliferative action of leflunomide’s active metabolite are not limited to its effects on pyrimidine synthesis. At higher doses, M1 inhibits tyrosine kinases responsible for early T cell and B cell signaling in the G_0_/G_1_ phase of the cell cycle (25). M1 has also been shown to inhibit phosphorylation of the Jak1 and Jak3 tyrosine kinases which are necessary for IL2R signaling (26) and inhibit activation of nuclear factor-κB (NF-κB) which is critical to the function of cells in the immune system (27).

Leflunomide is used in clinical practice as a disease-modifying antirheumatic drug. It has both anti-inflammatory and immunosuppressive characteristics. It is the first drug to receive the indication of retarding the structural damage of rheumatoid arthritis. The efficacy of leflunomide in the treatment of rheumatoid arthritis was shown in three controlled trials (28, 29). Leflunomide was shown to have significantly superior efficacy as compared to placebo, superior or similar efficacy to sulfasalazine and similar efficacy compared to methotrexate.

## **1.5 Leflunomide and HIV *in vitro***

Because of its unique ability to target and decrease proliferation of activated lymphocytes, leflunomide is an interesting potential therapeutic agent in the treatment of HIV infection. *In vitro* studies have already been completed examining the effect on HIV replication in PBMCs alone and in combination with antiretroviral agents. Its anti-HIV activity has also been studied in comparison to other immunomodulatory agents: hydroxyurea and mycophenolate mofetil.

Hossain et al infected PBMCs with wild-type HIV-1, an NRTI-resistant HIV-1 clone, and a lamivudine-resistant HIV-1 clone and then cultured them in varying amounts of M1, zidovudine and lamivudine (30). The authors found profound inhibition of wild-type HIV-1 infection in as little as 25 uM M1. When tested against the NRTI-resistant HIV-1 clone, M1 enhanced the antiviral effect of zidovudine by more than 20-fold. M1 also significantly inhibited the growth of the lamivudine-resistant HIV-1 clone and contributed to the inhibition of this clone by lamivudine in an additive manner.

Schläpfer et al examined the effect of M1 on HIV replication in PBMCs and tonsillar lymphocytes as compared to hydroxyurea and mycophenolate mofetil (31). All three drugs showed dose-dependent suppression of HIV replication with mycophenolate mofetil showing less potency than M1 or hydroxyurea. Uridine was added before or immediately after HIV infection and was able to restore infection when M1 was given at 25 or 50 umol/L but not at 100 umol/L.

M1 has also been shown to inhibit growth of cytomegalovirus (CMV) and herpes simplex virus (HSV) *in vitro*. Waldman et al have shown that CMV production in both human endothelial cells and fibroblasts is dramatically reduced in a dose-dependent manner over a range of pharmacologically relevant concentrations of M1 (32). It was found to be effective against multiple clinical isolates of CMV including multi-drug resistant isolates. The same group later showed that M1 has similar effects on HSV production in human endothelial cells and Vero cells (33).

## **1.6 Leflunomide and potential clinical use**

Clinical use of leflunomide is not without risks (see below section 9.3.1 Specific Medication Risk). There are potential risks of hepatotoxicity and pancytopenia as well as potential teratogenicity. However, if it is found to be effective in decreasing T cell turnover in HIV infected patients, it may prove useful as an adjuvant therapy in patients receiving non-hepatotoxic or myelosuppressive antiretroviral combinations, as a means of prolonging time to initiation of antiretroviral therapy, or as a means of prolonging interruptions in therapy. Furthermore, analogs of the leflunomide metabolite that have the same mechanism of action but have shorter half-lives and fewer toxicities are under development.

# 2. 0 Hypothesis

At 28 days, HIV-1 infected patients who receive leflunomide 20 mg daily will have lower levels of CD4+ T cell proliferation, as measured by mean percentage of CD4+ cells which express Ki67, compared to HIV-1 infected patients who receive placebo.

# 3.0 Study Objectives

## **3.1 Primary objective**

- To assess the effect of leflunomide on CD4+ T cell proliferation

## **3.2 Secondary objectives**

- To assess the effect of leflunomide on CD8+ T cell proliferation
- To assess the effect of leflunomide on T cell numbers
- To assess the effect of leflunomide on T cell activation
- To assess the effect of leflunomide on spontaneous apoptosis of T cells
- To assess the antiviral effect of leflunomide
- To assess the safety and tolerability of 20 mg of leflunomide for 28 days in HIV-1 infected patients

# 4.0 Study Design and Procedures

## **4.1 Design**

This is a randomized, double-blinded, placebo-controlled study to compare the effect of leflunomide versus placebo on CD4+ T cell proliferation in HIV-1 infected adults following 28 days of treatment. Patients who are determined to be eligible for enrollment will be randomized in a 2:1 ratio to receive leflunomide 20 mg orally, once daily or placebo. The study will consist of a screening period, a 28 day treatment period, and a 29 day post-treatment follow-up period which includes an 11 day drug-elimination period.

## **4.2 Endpoints**

**4.2.1 Primary endpoint**

- Change in % CD4+ cells expressing Ki67

## **4.2.2 Secondary endpoints**

- Change in total number of CD4+ cells expressing Ki67
- Change in total number and % CD8+ cells expressing Ki67
- Change in total and % CD4+ and CD8+ cells
- Change in total and % CD4+ and CD8+ cells expressing HLA-DR and CD38
- Change in total and % CD4+ and CD8+ cells which bind annexin V
- Change in plasma HIV viral load from baseline to end of treatment (baseline = average of H&P and day 1 measurements; End of treatment = day 29 measurement)
- Maximum change in plasma HIV viral load
- Frequency of toxicities and adverse events

# 4.3 Sample Size and Analysis

This study is designed to compare the change (day 28 less baseline) in the percentage of CD4 positive T cells expressing Ki67 between the two treatment groups. Randomization will be 2:1 favoring leflunomide.

Using historical data, a conservative estimate of the standard deviation of the change is 0.60 and a conservative estimate of the mean is 4.0. To detect a 30% drop in the mean to 2.8 from 4.0 with 90% power using a two-sided t-test with type 1 error rate set to 0.05 requires 10 patients in the leflunomide arm and 5 patients in the placebo arm.

With 1:1 randomization, 7 patients per arm are required to achieve the above parameters. However, with a 2:1 randomization, the standard error of the change in the leflunomide arm is reduced by 17% relative to a 1:1 randomization. Since the difference in total sample size for a 2:1 versus 1:1 randomization is minimal and precise estimation of the change in the leflunomide group desired, this study will use a 2:1 randomization. To allow for 20% dropout, 18 patients will be recruited: 12 in the leflunomide arm and 6 in the placebo arm. In order to enroll 18 patients, the total accrual ceiling will be 40, to account for patients who may screen but not enroll.

The primary analysis will be performed as treated using a t-test. Secondary analyses will be evaluated using tests and descriptive statistics, as appropriate. Analyses will also be performed as intent to treat. Assumptions for statistical procedures will be examined and appropriate alternatives will be used as necessary, e.g. log transformations or nonparametric methods.

#

## **4.4 Procedures**

**4.4.1 Screening period**

After signing screening informed consent, subjects will be evaluated in clinic to determine eligibility. At the time of the screening visit, the subject history will be reviewed and the following procedures will be performed (see Table 1):

- HIV testing
- Urine pregnancy testing for women of child-bearing potential (serum can be used for pregnancy testing in the event that urine is not collected)
- HIV viral load
- CD4+ and CD8+ T cell counts
- HLA testing
- Safety labs:
  - CBC with differential
  - acute care panel (sodium, potassium, chloride, bicarbonate, creatinine, urea nitrogen, creatinine)
  - hepatic panel (ALT, AST, alkaline phosphatase, total bilirubin, direct bilirubin)
  - mineral panel (albumin, calcium, magnesium, phosphorus)
  - amylase and lipase
  - PT and PTT
  - urinalysis
  - hepatitis screen (Hepatitis B surface antigen, anti-Hepatitis C antibody, hepatitis C PCR)
  - lipid panel (total cholesterol, triglycerides, HDL cholesterol, LDL cholesterol)
- EKG – (if patient can provide an EKG performed within 3 months of screening, the EKG will be deferred)
- CXR – (if patient can provide a CXR performed within 3 months of screening, the CXR will be deferred)

If the subject is determined to be eligible, he or she will be seen in clinic again for a history and comprehensive physical exam. The comprehensive physical exam includes weight, height, temperature, pulse, blood pressure, respiratory rate, pulse oximetry, and examination of the following:

- General appearance
- Skin
- Head, eyes, ears, nose, throat
- Neck
- Chest and lungs
- Heart
- Abdomen
- Extremities
- Cranial nerves, motor and sensory assessment

Blood will be drawn for repeat of safety labs (except hepatitis screen and lipids), HIV viral load and CD4+ and CD8+ T cell counts, urine will be sent for pregnancy testing and the subject will then sign the standard informed consent.

The screening period (time between signing screening informed consent and Day 1) will be no longer than 28 days.

After signing the standard informed consent, patients will be randomized to receive leflunomide or placebo. Randomization will be computer generated by the clinical center pharmacy staff. No one on the study team or the patient will have access to the randomization assignment until scheduled unblinding or earlier if clinically indicated for patient safety.

**4.4.2 Treatment period (Day 1 through 28)**

Patients must have stopped taking contraindicated medications one week prior to Day 1 (see Appendix A - Medications Metabolized by CYP 2C9 and Contraindicated During Treatment Period). Day 1 through 28, patients will receive leflunomide 20 mg orally, once daily or placebo. If appropriate, a urine pregnancy test will be performed on Day 1 before delivery of study drug.

On Days 1 and 15 of study, patients will return for clinic visits (it will be sufficient if patients come within plus or minus 2 days of each study visit during the treatment period and within plus or minus 7 days during the post-treatment follow up period). At these visits their history will be reviewed and they will undergo a targeted physical exam to be driven by any signs or symptoms identified by the patient. The targeted physical exam will also include temperature, pulse, blood pressure, respiratory rate and examination of the heart, lungs and abdomen. Patients will have urine sent for pregnancy testing if appropriate and blood drawn for safety labs, HIV viral load, CD4+ and CD8+ T cell counts, expression of Ki67, expression of activation markers, memory/naïve phenotyping, expression of coreceptors (CCR5 and CXCR4), ELISA for IL-7, TNF level, lymphoproliferative responses, Annexin V staining and *ex vivo* BrdU. Additionally, blood will be drawn for measurement of the main metabolite of leflunomide, M1 or A77 1726. On Day 1, blood will be drawn for these labs prior to administration of medication so that these values will serve as baseline values.

**4.4.3 Post-treatment follow up period (Day 29 through 64)**

On Day 29, the patient’s treatment arm will be unblinded. Patients who were receiving placebo will end study on Day 29. Patients who were receiving leflunomide will begin the drug elimination procedure: cholestyramine 8 grams orally three times a day for 11 days over the following 14 days. On Day 43, a serum A77 1726 level will be measured to assure elimination of the drug metabolite from the serum. Drug level should be less than 0.02 mg/L (or 0.02 ug/mL). If A77 1726 is less than 0.02 mg/L, the patient will end study on Day 64. If the level is above 0.02 mg/L, the 11 day cholestyramine elimination procedure will be repeated, and the study period will be extended as needed.

During the post-treatment follow up period, patients will come in for study visits on Days 29, 43 and 64. On these days, they will have their histories reviewed, urine will be sent for pregnancy testing when appropriate, and blood will be drawn for safety labs, HIV viral load, CD4+ and CD8+ T cell counts. On Days 29 and 43, blood will also be drawn for research studies of lymphocytes including expression of Ki67, expression of activation markers, memory/naïve phenotyping, expression of coreceptors (CCR5 and CXCR4), ELISA for IL-7, TNF level, lymphoproliferative responses, Annexin V staining and *ex vivo* BrdU as well as for measurement of A77 1726. On Days 29 and 43 they will undergo comprehensive physical exams and on Day 64 a targeted physical exam.

Table 1. Procedures

|  | Scr | H&P | D1^1^ | D15 | D29 | D43^2^ | D64^2^ |
| --- | --- | --- | --- | --- | --- | --- | --- |
| Screening consent | X |  |  |  |  |  |  |
| Standard consent |  | X |  |  |  |  |  |
| History | X | X | X | X | X | X | X |
| Comprehensive physical exam |  | X |  |  | X | X |  |
| Targeted physical exam |  |  | X | X |  |  | X |
| EKG and CXR | X |  |  |  |  |  |  |
| HLA testing | X |  |  |  |  |  |  |
| Safety labs^3^ | X | X | X | X | X | X | X |
| Urine pregnancy test^4^ | X | X | X | X | X | X | X |
| HIV ELISA^5^ | X |  |  |  |  |  |  |
| Hep B, C serology^6^ | X |  |  |  |  |  |  |
| Lipid panel | X |  |  |  |  |  |  |
| HIV viral load | X | X | X | X | X | X | X |
| Flow cytometry | X^7^ | X^7^ | X^8^ | X^8^ | X^8^ | X^8^ | X^7^ |
| Additional studies^9^ |  |  | X | X | X | X |  |
| A77 1726 level |  |  | X | X | X | X |  |

1. On Day 1, blood will be drawn prior to administration of study drug
2. Day 43 and 64 visits will occur only for patients who receive leflunomide
3. CBC with differential, acute care panel, hepatic and mineral panels, amylase, lipase, PT and PTT, urinalysis
4. Serum pregnancy testing can be used if urine is not collected
5. If ELISA positive, proceed with Western blot
6. Hepatitis B surface antigen, hepatitis C antibody, hepatitis C PCR
7. CD4+ and CD8+ T cell counts
8. CD4+ and CD8+ T cell counts, proliferation by intracellular Ki67 expression, expression of activation markers (CD25, CD38, HLA-DR), memory-naïve phenotyping, expression of CCR5 and CXCR4
9. ELISA for IL-7, TNF serum levels, lymphoproliferative responses, Annexin V staining, *ex vivo* BrdU

# 5. 0 Study Population

Eighteen HIV-1 infected patients with CD4+ cell count ≥350 cells/mm^3^ and HIV viral load ≥1,000 copies/mL who are not on any antiretroviral medications will be enrolled on this study.

# 5.1 Inclusion Criteria

1. Age > 18 and ≤ 65 years
2. HIV infected

1. CD4+ cell count ≥350 cells/mm^3^ at screening and historical nadir (since diagnosis of HIV) of ≥200 cells/mm^3^
2. Plasma HIV viral load ≥1,000 copies/mL at screening
3. Ability to understand and sign informed consent and willingness to comply with the study requirements and clinic policies
4. Must have a primary care physician who will be taking care of the patients for their HIV infection
5. Female study subjects of reproductive potential (defined as girls who have reached menarche or women who have not been post-menopausal for at least 24 consecutive months, i.e., who have had menses within the preceding 24 months or have not undergone a sterilization procedure (hysterectomy or bilateral oophorectomy), must have a negative serum or urine pregnancy test.
6. Agreement not to participate in a conception process (eg. active attempt to become pregnant or impregnate, sperm donation, or in vitro fertilization) and to follow strict contraceptive measures while on study.
7. If participating in sexual activity that could lead to pregnancy, the study volunteer must agree that two reliable methods of contraception will be used simultaneously during the entire study. Acceptable forms of contraception include:

- Condoms (male or female) with or without a spermicidal agent. Condoms are recommended because their appropriate use is the only contraception method effective for preventing HIV transmission.
- Diaphragm or cervical cap with spermicide
- IUD
- Hormonal-based contraception

Study subjects who are not of reproductive potential (women who have been post-menopausal for at least 24 consecutive months, women who have undergone hysterectomy or bilateral oophorectomy, or prepubescent boys or men who have documented azoospermia) are eligible without requiring the use of contraception. Written or oral documentation communicated by clinician or clinician’s staff of one of the following:

- Physician report/letter.
- Operative report or other source documentation in the patient record (a laboratory report of azoospermia is required to document successful vasectomy).
- Discharge summary.
- Laboratory report of azoospermia.
- FSH measurement elevated into the menopausal range as established by the reporting laboratory.

# 5.2 Exclusion Criteria

1. Current treatment with antiretrovirals or use of antiretrovirals within 12 weeks of screening
2. Intention to start antiretroviral regimen within 64 day study period
3. Previous treatment with leflunomide
4. Previous treatment with IL-2
5. Treatment with immunomodulatory agents (including hydroxyurea, mycophenolate, cyclosporine, rapamycin, anti-HIV vaccines, interleukins other than IL-2, interferons) within 60 days of study
6. Treatment with systemic corticosteroids within 30 days of study
7. Inability or unwillingness to discontinue drugs (except NSAIDs) that are metabolized by P450 2C9 isoenzyme (see Appendix A - Medications Metabolized by CYP 2C9 and Contraindicated During Treatment Period).
8. Inability or unwillingness to discontinue hepatotoxic drugs (eg. isoniazid, rifampin, HMG CoA reductase inhibitors)
9. Inability or unwillingness to discontinue drugs that interact with cholestyramine (eg. have diminished absorption with cholestyramine) (see Appendix B – Medications that Have Interactions with Cholestyramine)
10. Current use of or known intolerance of cholestyramine or bile acid sequestering resins
11. History of familial hyperlipoproteinemia type III, IV or V
12. Active bacterial infection within 4 weeks of screening
13. History of AIDS-defining illness (category C) (see Appendix C - CDC AIDS Classification Criteria)
14. Hepatitis B or C infection
15. Acute or chronic liver disease from any cause (eg. alcoholic hepatitis or alcohol induced cirrhosis, autoimmune hepatitis, primary biliary cirrhosis, sclerosing cholangitis, Wilson’s disease, hemochromatosis) which the investigator feels would compromise the subject’s safety
16. History of biliary obstruction
17. Current alcohol abuse or unwillingness to abstain from alcohol use for study period
18. History of hypertension that is not controlled (<140/80 mm/Hg) on a single antihypertensive agent
19. Abnormal laboratory findings: hemoglobin <10 g/dL; ANC <1000/mm^3^; platelets <100,000/mm^3^; creatinine above the upper limit of normal; ALT or alkaline phosphatase >1.25 times the upper limit of normal; AST >1.25 times the upper limit of normal* direct bilirubin >1.5 times the upper limit of normal; total bilirubin >2 times the upper limit of normal lipase > 1.5 times the upper limit of normal; PT >1.1 times the upper limit of normal; PTT>1.5 times the upper limit of normal.
20. History of malignant neoplasm except in situ anogenital carcinoma, adequately treated basal or squamous cell carcinoma of the skin or solid tumors treated with curative therapy and disease free for at least five years
21. Significant medical or psychiatric disorder which the investigator feels would interfere with the subject’s ability to participate or would compromise safety
22. Women who are currently pregnant or breast-feeding
23. History of interstitial lung disease

* Subjects with isolated AST elevation, higher than normal CPK values in the absence of liver disease and compatible history such as intense exercise will be allowed to re-screen if the study investigators suspect that the elevated AST is of muscular origin.

# 6.0 Patient Monitoring and Criteria for Withdrawal from Study

**6.1 Patient monitoring**

Monitoring will be performed by the principal investigator and study team. During the treatment and post treatment follow up periods, a review of the patient’s medical history will be performed at the NIH at each scheduled follow up visit and either a complete or targeted physical exam (Table 1). After screening and prior to signing the standard consent, patients will undergo a complete physical exam by a staff physician. Additionally, they will undergo a complete physical exam by a staff physician on Days 29 and 43. On Days 1, 15 and 64, targeted physical exams will be performed as clinically indicated. Evaluations will also take place more frequently as clinically indicated. Laboratory monitoring at these visits will include the following:

- CBC with differential, acute care panel, hepatic and mineral profiles, amylase, lipase, PT and PTT, urinalysis, and urine pregnancy testing (if appropriate)
- CD4+ and CD8+ T cell counts
- HIV plasma viral load
- Expression of Ki67, CD25, HLA-DR, CD38
- Memory/naïve phenotyping of lymphocytes
- A77 1726 level

Blood will be obtained for additional research purposes. Studies to be done include the following:

- ELISA for IL-2, IL-7, TNFα serum levels
- Lymphoproliferative responses
- Annexin V staining
- *Ex vivo* BrdU

6.2 A77 1726 levels

During the treatment and follow up period, blood will also be obtained for measurement of A77 1726, the main metabolite of leflunomide. These levels will be used to correlate with primary and secondary endpoints as well as to assure complete clearance of the metabolite at the end of the elimination procedure. The levels will be measured by Labcorp Clinical Trials Department. Levels that are drawn during the treatment period will be stored and run in batches so as not to unblind patient treatment arms. However, the level that is drawn following the elimination procedure will be measured in real time so that a decision can be made as to whether that patient will need to repeat the elimination procedure.

**6.3 Criteria for stopping study drug and immediate institution of drug elimination procedure**

If a patient meets any of the following criteria for stopping study drug, his dosing assignment will be unblinded and if he was receiving leflunomide, he will be started immediately on the drug elimination procedure:

1. Patient develops malignancy or serious infection
2. Patient becomes pregnant
3. Patient develops elevation of liver enzymes greater than 2X the upper limit of normal (if liver enzymes are ≥3 X the upper limit of normal the study drug will be stopped immediately; if liver enzymes are >2 but <3 X the upper limit of normal, they will be repeated in 24 to 72 hours and if they remain >2 X the upper limit of normal the study drug will be stopped)
4. Patient develops grade III or IV adverse event or toxicity
5. Patient develops grade II or greater allergic reaction or urticaria
6. Patient develops a drop in CD4+ T cell count of ≥40% of baseline or a CD4+ T cell count of <200 that is confirmed with a second measurement (after 10-14 days) [Baseline CD4+ T cell count = average of CD4+ T cell count at H&P and Day 1]
7. Patient develops a medical condition that in the opinion of the investigator indicates that discontinuing the study drug is in the subject’s best interest

Additional criteria for stopping study drug:

1. It is felt by the principal investigator to be in the patient’s best interest, even if the patient does not agree
2. The patient begins taking any of the contraindicated medications listed in Appendix A or B or any other medication listed as an exclusion criteria and does not agree to discontinue it
3. The patient begins taking antiretroviral medications
4. Patient desire to leave study
5. Patient noncompliance
6. Termination of study

At the time of withdrawal from study, a member of the study team (the study coordinator, principal investigator, associate investigator or covering physician) will contact the pharmacy for unblinding of treatment arm (PDS during business hours Monday through Friday or inpatient pharmacy after hours and on weekends). If a patient was receiving leflunomide, oral cholestyramine 8 g three times daily will be administered for a total of 11 days. Serum A77 1726 level will be measured upon completion of this procedure. If the A77 1726 level is above 0.02 mg/L, the elimination procedure will be repeated.

In addition, safety and research labs will be performed as at other clinic visits. Any other testing will be performed as clinically indicated.

If a patient ends study due to a drop in CD4+ T cell count, the patient’s CD4+ T cell count will be monitored following the leflunomide elimination procedure. If the CD4+ T cell count remains decreased 10 – 14 days after the elimination procedure is completed, the patient will be referred to their primary care physician for further monitoring and consideration of treatment with antiretrovirals, depending on their actual CD4+ T cell count and viral load.

If patients withdraw from study prior to receiving study drug, they will be replaced by enrolling new patients in their place. This refers only to patients who withdraw prior to receiving study drug and whose treatment arm has not been unblinded to either the subject or the study team.

If a patient withdraws from study for any reason, every effort will be made to attain complete data from that patient, including endpoint data at Day 29.

6.4 Criteria for stopping enrollment

A Data Safety Monitoring Board (DSMB) will meet at a predetermined time (when 8 subjects have completed the 28 day active treatment phase) as well as on an urgent basis if 2 or more patients meet criteria for stopping study drug due to adverse events that are possibly, probably, or definitely related to the study drugs. This board will review safety data and determine whether the study should continue enrollment and/or study drug dosing. If enrollment is closed and the study terminated, the dosing arm of those patients who are enrolled at that time and receiving the study drug will be unblinded. As soon as possible, a member from the pharmacy will inform the study coordinator of the patient’s treatment arm, and the study coordinator will inform the patient. Those patients who had been receiving leflunomide will undergo the drug elimination procedure.

**7.0 Adverse Event Reporting**

**7.1 Definitions**

Adverse events (AE): Any unfavorable and unintended diagnosis, symptom, sign (including an abnormal laboratory finding), syndrome or disease which either occurs during the study, having been absent at baseline, or if present at baseline, appears to worsen.

Serious adverse events (SAE): Any untoward medical occurrence that

- 1. results in death
  2. is life-threatening
  3. requires hospitalization or prolongation of existing hospitalization
  4. results in disability/incapacity
  5. results in congenital anomaly/birth defect
  6. other situations such as important medical events (eg. diagnosis of cancer) that may not be immediately life-threatening or result in death or hospitalization but may jeopardize the subject or may require medical or surgical intervention to prevent one of the other outcomes listed above

Unexpected adverse event: An adverse event that is not described in the package insert or current literature with regards to leflunomide or cholestyramine

Relationship assessment: The relationship between a serious adverse event and study drug is determined by the principal investigator. The degree of certainty about causality is graded as follows:

- Unrelated: adverse event is clearly due to extraneous causes
- Unlikely (must have two): adverse event:
  - Does not have temporal relationship to intervention
  - Could readily have been produced by the subject’s clinical state
  - Could have been due to environmental or other interventions
  - Does not follow known pattern of response to intervention
  - Does not appear to worsen with reintroduction of intervention
- Possible (must have 2): adverse event:
  - Has a reasonable temporal relationship to intervention
  - Could not readily have been produced by the subject’s clinical state
  - Could not readily have been due to environmental or other interventions
  - Follows a known pattern of response to intervention
- Probable (must have 3): adverse event
  - Has a reasonable temporal relationship to intervention
  - Could not readily have been produced by the subject’s clinical state or have been due to environmental or other interventions
  - Follow a known pattern of response to intervention
  - Disappears or decreases with reduction in dose or cessation of intervention
- Definite (must have all 4): adverse event
  - Has a reasonable temporal relationship to intervention
  - Could not readily have been produced by the subject’s clinical state or have been due to environmental or other interventions
  - Follows a known pattern of response to intervention
  - Disappears or decreases with reduction in dose or cessation of intervention

## **7.2 Reporting**

Adverse events: Expected adverse events are those listed in the package inserts for leflunomide and cholestyramine. Adverse events, as well as degree of relationship to study drug, will be entered into a database system; for this system they are graded according to the AIDS Clinical Trial Group (ACTG) Toxicity Table (See Appendix D). For abnormalities NOT found elsewhere on the Toxicity Table they will be assessed for severity:

- Grade 1: Transient or mild discomfort; no limitation in activity; no medical intervention/therapy required.
- Grade 2: Mild to moderate limitation in activity - some assistance may be needed; no or minimal medical intervention/therapy required.
- Grade 3: Marked limitation in activity, some assistance usually required; medical intervention/therapy required, hospitalization possible.
- Grade 4: Extreme limitation in activity, significant assistance required; significant medical intervention/therapy required, hospitalization or hospice care.

7.2.1 Reporting to the IRB

The following types of serious adverse events, regardless of relationship to study drug, will be reported to the IRB by fax or phone within 24 hours of awareness of the event followed by written SAE form within 3 days of awareness of event:

- Death
- Life-threatening event
- Cancer occurring on study
- Pregnancy
- Congenital anomaly/birth defect
- Permanent disability/incapacity

The following types of serious adverse events will be reported to the IRB by written SAE form within 3 days of knowledge of the event:

- Grade 3 and 4 adverse events
- Hospitalizations
- Adverse events of any toxicity considered serious by the principle investigator
- Any other event or condition which in the judgment of the principle investigator represents a significant hazard

The following types of adverse events will be reported to the IRB at annual review of the protocol:

- Grade 2 adverse events except those determined to be unrelated or unlikely to be related to study drug (Grade 3 and 4 adverse events will be reported as described above)

7.2.2 Reporting to the sponsor

NIAID is the sponsor for the Investigational New Drug (IND) application filed at the US Food and Drug Administration (FDA). In accordance with the Code of Federal Regulations (CFR), the IND sponsor and investigators participating in a clinical trial are responsible for the proper reporting of serious adverse events (SAEs). The purpose of reporting SAEs is to better understand the toxicity and safety of investigational agents. SAE reporting and monitoring also assist in alerting the FDA, sponsor, and clinical investigators of real and potential patient safety issues. NIAID must report SAEs that are serious, unexpected, and related to the study drug to the FDA in the form of a written IND Safety Report. In general, IND Safety Reports must be submitted to the FDA as soon as possible, but no later than 15 calendar days after the sponsor is notified of the SAE. Deaths and life-threatening events with any possible relationship to a study drug must be reported to the FDA within 7 calendar days of the sponsor’s awareness.

All deaths and life-threatening SAEs will be reported to Regulatory Compliance and Human Subjects Protection Branch (RCHSPB) Safety Department within 1 business day, and all other SAEs will be reported within 3 business days. The Safety Department can be contacted by phone at 301-846-5301, by fax at 301-846-6224, and by email at [rchspsafety@mail.nih.gov](mailto:rchspsafety@mail.nih.gov). RCHSPB will report SAEs for the FDA in accordance with 21 CFR 312.

# 8.0 Protocol Monitoring

The trial will be conducted in compliance with this protocol, International Conference on Harmonization (ICH) Guideline for Good Clinical Practices (GCP) and any applicable regulatory requirements. Monitors under contract with the RCHSPB will visit the clinical research site to monitor all aspects of the study in accordance with the appropriate regulations. The objectives of a monitoring visit will be: 1) to verify the prompt reporting of all data points, including reporting SAEs and checking availability of signed informed consent, 2) to compare individual subject records, CRIMSON data pulls and the source documents (supporting data, laboratory specimen records and medical records to include physician progress notes, nurses’ notes, subjects’ hospital charts), 3) to ensure protection of study subjects, compliance with the protocol, and accuracy and completeness of records. The monitors will also inspect the clinical site regulatory files to ensure that regulatory requirements (FDA/OHRP/ICH-GCP) are being followed. During the monitoring visits, the principal investigator (and/or designee) and other study personnel will be available to discuss the study progress and monitoring visit.

The principal investigator (and/or designee) will make study documents (eg. consent forms, CRIMSON data pulls) and pertinent hospital or clinical records readily available for inspection by the local IRB, the FDA, the site monitors, and the NIAID staff for confirmation of the study data.

**8.1 Data Safety Monitoring Board**

The NIAID Data Safety Monitoring Board (DSMB) will be asked to monitor the progress of the study while still blinded to the investigators. The NIAID Intramural DSMB is constituted to review the safety data of all NIAID-sponsored clinical studies that require DSMB oversight and consists of experts in infectious diseases, biostatistics, and clinical trials. The DSMB will review cumulative study data twice per year to evaluate safety, study conduct, and scientific validity and integrity of the trial. As part of this responsibility, DSMB members must be satisfied that the timeliness, completeness, and accuracy of the data submitted to them for review are sufficient for evaluation of the safety and welfare of study participants. The DSMB will also assess the performance of the overall study operations and any other relevant issues as necessary.

In addition to the biannual meetings, the DSMB will meet to review the data when 8 patients (47% of total expected enrollment) have been enrolled and have completed the treatment period. The group will also convene on an urgent basis if two or more subjects meet criteria for stopping study drug (see **section 6.3**) due to toxicities.

# 9.0 Human Subjects Protection

**9.1 Subject selection criteria**

Patients will be recruited for participation in this study without bias to gender, racial, economic, or social status. Patients new to the NIH will be recruited from outside physician referrals. Subjects will be recruited from NIH clinics and local public, academic, and private clinics. The study will be advertised and posted on the NIH web site.

The safety of leflunomide in the pediatric population has not been studied, and its use is not recommended in children. Therefore children (age <18 years) will be excluded from this study.

**9.2 Benefits**

There are no immediate clinical benefits for patients who participate in this study. However, the study will make a significant contribution to the knowledge of HIV immunopathogenesis with potential therapeutic implications for the future.

**9.3 Risks**

The potential risks of this study are:

- It is possible that treatment with leflunomide will lead to more adverse events or fluctuation in HIV viral load
- Risks of the medications: leflunomide and cholestyramine, which are discussed below

**9.3.1 Specific medication risk**

**9.3.1.1 Leflunomide**

As outlined in the package insert (submitted separately), the safety of leflunomide was examined in three clinical trials that enrolled a total of 1839 patients with active rheumatoid arthritis of whom 816 received leflunomide and 1023 received either placebo, methotrexate, or sulfasalazine. Patients in these clinical trials received an initial loading dose of leflunomide of 100 mg per day for 3 days followed by maintenance therapy with 20 mg/day.

Leflunomide is contraindicated in two patient populations: 1) patients with known hypersensitivity to leflunomide, and 2) women who are or may become pregnant. The package insert contains a box warning that states pregnancy must be excluded before the start of treatment with leflunomide, and pregnancy must be avoided during leflunomide treatment or prior to the completion of the drug elimination procedure after leflunomide treatment.

Leflunomide can cause fetal harm when administered to a pregnant woman. When administered orally to rats during organogenesis at a dose of 15 mg/kg, leflunomide was teratogenic (most notably anophthalmia or microophthalmia and internal hydrocephalus). The systemic exposure of rats at this dose was approximately 1/10 the human exposure level based on AUC. Under these exposure conditions, leflunomide also caused a decrease in the maternal body weight and an increase in embryo lethality with a decrease in fetal body weight for surviving fetuses. In rabbits, oral treatment with 10 mg/kg of leflunomide during organogenesis resulted in fused, dysplastic sternebrae. The exposure level at this dose was essentially equivalent to the maximum human exposure level based on AUC. At a 1 mg/kg dose, leflunomide was not teratogenic in rats or rabbits.

When female rats were treated with 1.25 mg/kg of leflunomide beginning 14 days before mating and continuing until the end of lactation, the offspring exhibited marked (greater than 90%) decreases in postnatal survival. The systemic exposure at 1.25 mg/kg was approximately 1/100 the human exposure level based on AUC.

The package insert for leflunomide also contains several warnings:

- Immunosuppression Potential/Bone Marrow Suppression: leflunomide is not recommended in persons with severe immunodeficiency, bone marrow dysplasia, or severe uncontrolled infections. Rarely, severe infections including sepsis, which may be fatal, have been reported in patients receiving leflunomide. Most of the reports were confounded by concomitant immunosuppressant therapy and/or comorbid illness which, in addition to rheumatoid disease, may predispose patients to infection.

There have been rare reports of pancytopenia, agranulocytosis, and thrombocytopenia in patients receiving leflunomide alone. These events have been reported most frequently in patients who received concomitant treatment with methotrexate or other immunosuppressive agents, or who had recently discontinued these therapies; in some cases, patients had a prior history of a significant hematologic abnormality.

- Hepatotoxicity: Rare cases of severe liver injury, including with fatal outcome, have been reported during treatment with leflunomide. Most cases of severe liver injury occur within 6 months of therapy and in a setting of multiple risk factors for hepatotoxicity (liver disease, other hepatotoxins).

In clinical trials, leflunomide treatment as monotherapy or in combination with methotrexate was associated with elevations of liver enzymes, primarily ALT and AST, in a significant number of patients; these effects were generally reversible. Most transaminase elevations were mild (≤2-fold the upper limit of normal) and usually resolved while continuing treatment. Marked elevations (>3-fold the upper limit of normal) occurred infrequently and reversed with dose reduction or discontinuation of treatment.

- Pre-existing hepatic disease: Given the possible risk of increased hepatotoxicity, and the role of the liver in drug activation, elimation and recycling, the use of leflunomide is not recommended in patients with significant hepatic impairment or evidence of infection with hepatitis B or C viruses.
- Skin reactions: Rare cases of Stevens-Johnson syndrome and toxic epidermal necrolysis have been reported in patients receiving leflunomide.
- Malignancy: The risk of malignancy, particularly lymphoproliferative disorders, is increased with the use of some immunosuppression medications. There is a potential for immunosuppression with leflunomide. No apparent increase in the incidence of malignancies and lymphoproliferative disorders was reported in the clinical trials of leflunomide, but larger and longer-term studies would be needed to determine whether there is an increased risk of malignancy or lymphoproliferative disorders with leflunomide.

The package insert also contains the following precaution:

- Respiratory: Interstitial lung disease has been reported during treatment with lefunomide and has been associated with fatal outcomes. Interstitial lung disease is a potentially fatal disorder, which may occur acutely at any time during therapy and has a variable clinical presentation. New onset or worsening of pulmonary symptoms, such as cough and dyspnea, with or without associated fever, may be a reason for discontinuation of therapy and for further investigation as appropriate.

Adverse reactions associated with the use of leflunomide in rheumatoid arthritis include diarrhea, elevated liver enzymes, alopecia and rash. In the controlled studies, the following adverse events were reported, regardless of causality: allergic reaction (1-5%), asthenia (3-6%), flu syndrome (0-4%), infection (0-4%), injury accident (5-7%), pain (1-4%), abdominal pain (5-6%), back pain (5-8%), hypertension (9-10%), chest pain (1-2%), anorexia (3%), diarrhea (17-27%), dyspepsia (5-10%), gastroenteritis (1-3%), abnormal liver enzymes (5-10%), nausea (9-13%), mouth ulcer (3-5%), vomiting (3-5%), hypokalemia (1-3%), weight loss (2-4%), arthralgia (1-4%), leg cramps (0-4%), joint disorder (2-8%), synovitis (1-4%), tenosynovitis (2-5%), dizziness (4-7%), headache (7-13%), paresthesia (2-4%), bronchitis (5-8%), increased cough (3-5%), respiratory infection (15-27%), pharyngitis (2-3%), pneumonia (2-3%), rhinitis (2-5%), sinusitis (1-5%), alopecia (9-17%), eczema (1-3%), pruritus (4-6%), rash (10-12%), dry skin (2-3%), urinary tract infection (5%).

In addition the following side effects have been reported in 1% to <3% of the rheumatoid arthritis patients in the leflunomide treatment group in controlled trials.

- Body as a whole: abscess, cyst, fever, hernia, malaise, pain, neck pain, pelvic pain
- Cardiovascular: angina pectoris, migraine, palpitation, tachycardia, varicose vein, vasculitis, vasodilation
- Gastrointestinal: cholelithiasis, colitis, constipation, esophagitis, flatulence, gastritis, gingivitis, melena, oral moniliasis, pharyngitis, salivary gland enlarged, stomatitis (or aphthous stomatitis), tooth disorder
- Endocrine: diabetes mellitus, hyperthyroidism
- Hemic and lymphatic system: anemia (including iron deficiency anemia), ecchymosis
- Metabolic and nutritional: creatinine phosphokinase increased, hyperglycemia, hyperlipidemia, peripheral edema
- Musculo-skeletal system: arthrosis, bone necrosis, bone pain, bursitis, muscle cramps, myalgia, tendon rupture
- Nervous system: anxiety, depression, dry mouth, insomnia, neuralgia, neuritis, sleep disorder, increased sweating, vertigo
- Respiratory system: asthma, dyspnea, epistaxis, lung disorder
- Skin and appendages: acne, contact dermatitis, fungal dermatitis, hair discoloration, hematoma, herpes simplex, herpes zoster, maculopapular rash, nail disorder, skin discoloration, skin disorder, skin nodule, subcutaneous nodule, skin ulcer
- Special senses: blurred vision, cataract, conjunctivitis, eye disorder, taste perversion
- Urogenital system: albuminuria, cystitis, dysuria, hematuria, menstrual disorder, prostate disorder, urinary frequency, vaginal moniliasis

Other less common adverse events seen in clinical trials include: 1 case of anaphylactic reaction occurred following restarting of drug after withdrawal due to rash; urticaria, increased eosinophil count, transient low platelet count, and low white bood cell count.

Since the licensing of leflunomide for public use, the following side effects have been reported rarely.

- Body as a whole: opportunistic infections, severe infections including sepsis that may be fatal;
- Gastrointestinal: pancreatitis
- Hematologic: low white blood cell count, low neutrophil count, low platelet count
- Hypersensitivity: angioedema
- Hepatic: hepatitis, jaundice, severe liver injury such as hepatic failure and acute hepatitic necrosis that may be fatal
- Respiratory: interstitial lung disease, including interstitial pneumonitis and pulmonary fibrosis, which may be fatal
- Nervous system: peripheral neuropathy
- Skin and appendages: erythema multiforme, Stevens-Johnson syndrome, toxic epidermal necrolysis

No evidence of carcinogenicity was observed in a 2-year bioassay in rats at oral doses of leflunomide up to the maximally tolerated dose of 6 mg/kg (approximately 1/40 the maximum human M1 systemic exposure based on AUC). However, male mice in a 2-year bioassay exhibited an increased incidence in lymphoma at an oral dose of 15 mg/kg, the highest dose studied (1.7 times the human M1 exposure based on AUC). Female mice in the same study exhibited a dose-related increased incidence of bronchoalveolar adenomas and carcinomas combined beginning at 1.5 mg/kg (approximately 1/10 the human M1 exposure based on AUC). The significance of the findings in mice relative to the clinical use of leflunomide is not known.

Leflunomide was not mutagenic in the Ames Assay, the Unscheduled DNA Synthesis Assay, or in the HGPRT Gene Mutation Assay. In addition, leflunomide was not clastogenic in the *in vivo* Mouse Micronucleus Assay nor in the *in vivo* Cytogenetic Test in Chinese Hamster Bone Marrow Cells. However, 4-trifluoromethylaniline (TFMA), a minor metabolite of leflunomide, was mutagenic in the Ames Assay and in the HGPRT Gene Mutation Assay, and was clastogenic in the *in vitro* Assay for Chromosome Aberrations in the Chinese Hamster Cells. TFMA was not clastogenic in the *in vivo* Mouse Micronucleus Assay nor in the *in vivo* Cytogenetic Test in Chinese Hamster Bone Marrow Cells.

**9.3.1.2 Cholestyramine**

Cholestyramine is a bile acid sequestrant and is used in the leflunomide elimination procedure. When given at a dose of 8 g three times a day for 24 hours to healthy volunteers, cholestyramine decreased the plasma levels of A77 1726 by 40% at 24 hours and by 49-65% in 48 hours.

As indicated in the package insert (submitted separately), use of cholestyramine is contraindicated in patients with complete biliary obstruction, hyperlipoproteinemia types III, IV, or V, or known hypersensitivity to bile acid sequestering resins.

The most common adverse reactions caused by cholestyramine are gastrointestinal including constipation, abdominal discomfort, nausea, flatulence, vomiting, diarrhea, heartburn, anorexia, and indigestion. Mild steatorrhea may also occur. Constipation is the most common complaint of cholestyramine users. Cholestyramine should be used with caution in patients with constipation at baseline, and routine use of a stool softener should be considered with long term use of cholestyramine. Complete intestinal obstruction has been reported in a couple of patients.

Other adverse effects of cholestyramine include rare reports of hematologic effects. Bleeding tendencies due to hypoprothrombinemia have been reported as well as one case of platelet gigantism.

Hyperchloremic acidosis has been reported to occur in small children and older adults receiving concomitant spironolactone therapy.

Elevated thyrotropin levels with a corresponding reduction in serum free thyroxine values occurred in a few hypothyroid patients who were receiving thyroid hormone and cholestyramine concurrently. It is unknown if the alteration in the thyroid function tests were due to a drug interaction or an alteration of thyroid function.

Cholestyramine may interfere with normal fat absorption and thus prevent absorption of fat soluble vitamins A, D, E, and K.

Sipping or holding the resin in the mouth for prolonged periods may lead to changes in the surface of the teeth resulting in discoloration, erosion of enamel or decay.

There is one reported case of urinary tract calculi associated with cholestyramine therapy.

Cholestyramine may interfere with drugs that undergo enterohepatic circulation. Concomitant drugs should be taken an hour before or 4-6 hours after taking cholestyramine. There are additive effects with cholestyramine and HMG-CoA reductase inhibitors and nicotinic acid. Cholestyramine may reduce of delay absorption of several medications (see Appendix B – Medications that Have Interactions with Cholestyramine)

**9.3.2 Other Risks**

Blood drawing has associated risks that include bruising, bleeding, and rarely fainting or infection. No more then 450 cc of blood will be drawn during a 6-week period in accordance with NIH guidelines.

This research study involves exposure to radiation from a chest x-ray, which is for research purposes only.  The amount of radiation involved in this procedure confers minimal risk and is necessary to obtain the research information desired. A chest X-ray involves exposure of 100 mrem to the skin of the chest and the calculated effective dose is 29 mrem. The amount of radiation exposure in this study is below the dose guideline established by the NIH Radiation Safety Committee for research subjects.  This guideline is an effective dose of 5 rem (or 5,000 mrem) received per year.

# 9.4 Compensation

There will be financial compensation of $640.00 for those who enroll and complete the entire study. Participants will also receive partial remuneration for the immediate costs associated with their study-related expenses like travel expenses, lodging, etc., as provided by the NIH.

In the event of an injury occurring as a result of participation in this research study, participants will be advised to seek immediate necessary medical care from their home physician. There is no provision for long term free medical care or for monetary compensation from any injury from the physicians conducting this study, from the National Institute of Allergy and Infectious Diseases (NIAID) or from the Clinical Center of the National Institutes of Health.

# 9.5 Consent/Assent Procedures

The protocol will be discussed verbally between the patient and one of the senior investigators at OP8 during which time the patient will have ample opportunity to discuss any questions. The patient will then be given the opportunity for discussion with one of the senior investigators. If protocol enrollment is then agreeable, the patient will sign the consent document and treatment will commence.

9.6 Confidentiality

All laboratory specimens, evaluation forms, reports, and other records that leave the site will be identified by coded number only in order to maintain subject confidentiality. All records will be kept locked. All computer entry and networking programs will be done with coded numbers only. Clinical information will not be released without written permission of the subject, except as necessary for monitoring by IRB, the FDA, and the NIAID.

**10.0 References**

1. Grossman, Z., Meier-Schellersheim, M., Sousa, A.E., et al. CD4+ T cell depletion in HIV infection: are we closer to understanding the cause? Nat Med 2002; 8: 319-323.

2. McCune, J.M. The dynamics of CD4+ T-cell depletion in HIV disease. Nature 2001; 410: 974-979.

3. Sousa, A.E., Carneiros, J., Meier-Schellersheim, M., et al. CD4 T cell depletion is linked directly to immune activation in the pathogenesis of HIV-1 and HIV-2 but only indirectly to the viral load. J Immunol 2002; 169: 3400-3406.

4. Anthony, K.B., Yoder, C., Metcalf, J.A., et al. Incomplete CD4 T cell recovery in HIV-1 infection after 12 months of highly active antiretroviral therapy is associated with ongoing increased CD4 T cell activation and turnover. J Acquir Immune Defic Syndr 2003; 33: 125-133.

5. Liu, Z., Cumberland, W.G., Hultin, L.E., et al. Elevated CD38 antigen expression on CD8+ T cells is a stronger marker for the risk of chronic HIV disease progression to AIDS and death in the Multicenter AIDS Cohort Study than CD4+ cell count, soluble immune activation markers, or combinations of HLA-DR and CD38 expression. J Acquir Immune Defic Syndr Hum Retrovirol 1997; 16: 83-92.

6. Hazenberg, M.D., Otto, S.A., van Benthem, B.H., et al. Persistent immune activation in HIV-1 infection is associated with progression to AIDS. AIDS 2003; 17: 1881-1888.

7. Mohri, H., Perelson, A.S., Tung, K., et al. Increased turnover of T lymphocytes in HIV-1 infection and its reduction by antiretroviral therapy. J Exp Med 2001; 194: 1277-1287.

8. Mohri, H., Bonhoeffer, S., Monard, S., et al. Rapid turnover of T lymphocytes in SIV-infected rhesus macaques. Science 1998; 279: 1223-1227.

9. Kovacs, J.A., Lempicki, R.A., Sidorov, I., et al. Identification of dynamically distinct subpopulations of T lymphocytes that are differentially affected by HIV. J Exp Med 2001; 194: 1731-1741.

10. Silvestri, G., and M.B. Feinberg. Turnover of lymphocytes and conceptual paradigms in HIV infection. J Clin Invest 2003; 112: 821-824.

11. Gao, W.Y., Cara, A., Gallo, R.C., et al. Low levels of deoxynucleotides in peripheral blood lymphocytes: a strategy to inhibit human immunodeficiency virus type 1 replication. Proc Natl Acad Sci U. S. A. 1993; 90: 8925-8928.

12. Malley, S.D., Grange, J.M., Hamedi-Sangsari, F., et al. Synergistic anti-human immunodeficiency virus type 1 effect of hydroxamate compounds with 2’, 3’-dideoxyinosine in infected resting human lymphocytes. Proc Natl Acad Sci U. S. A. 1994; 91: 11017-11021.

13. Biron, F., Lucht, F., Peyramond, D., et al. Anti-HIV activity of the combination of didanosine and hydroxyurea in HIV-1-infected individuals. J Acquir Immune Defic Syndr Hum Retrovirol 1995; 10: 36-40.

14. Simonelli, C., Nasti, G., Vaccher, E., et al. Hydroxyurea treatment in HIV-infected patients. J Acquir Immune Defic Syndr Hum Retrovirol 1996; 13: 462-464.

15. Chapuis, A.G., Rizzardi, G., D’Agnostino, C., et al. Effects of mycophenolic acid on human immunodeficiency virus in vitro and in vivo. Nat Med 2000; 6: 762-768.

16. Coull, J.J., Turner, D., Melby, T., et al. A pilot study of the use of mycophenolate mofetil as a component of therapy for multidrug-resistant HIV-1 infection. J Acquir Immune Defic Syndr 2001; 26: 423-434.

17. Margolis, D.M., Kewn, S., Coull, J.J., et al. The addition of mycophenolate mofetil to antiretroviral therapy including abacavir is associated with depletion of intracellular deoxyguanosine triphosphate and a decrease in plasma HIV-1 RNA. J Acquir Immune Def 2002; 31: 45-49.

18. Andrieu, J.M., Lu, W., and R. Levy. Sustained increases in CD4 cell counts in asymptomatic human immunodeficiency virus type 1-seropositive patients treated with prednisolone for 1 year. J Infect Dis 1995; 171: 523-530.

19. Andrieu, J.M., and W. Lu. Long-term clinical, immunologic and virologic impact of glucocorticoids on the chronic phase of HIV infection. BMC Med 2004; 2: only electronic for now.

20. Miller, K.D., Masur, H., Jones, E.C., et al. High prevalence of osteonecrosis of the femoral head in HIV-infected adults. Ann Int Med 2002; 137: E-17 – E-25.

21. Wallis, R.S., Kalayjian, R., Jacobson, J.M. et al. A study of the immunology, virology, and safety of prednisone in HIV-1 infected subjects with CD4 cell counts of 200 to 700 mm3. J Acquir Immune Defic Syndr 2003; 32: 281-286.

22. Quemeneur, L., Gerland, L., Flacher, M., et al. Differential control of cell cycle, proliferation, and survival of primary T lymphocytes by purine and pyrimidine nucleotides. J Immunol 2003; 170: 4986-4995.

23. Rozman, B. Clinical pharmacokinetics of leflunomide. Clin Pharmacokinet 2002; 41: 421-430.

24. Fox, R.I. Mechanism of action of leflunomide in rheumatoid arthritis. J Rheumtol Suppl 1998; 25: 20-26.

25. Nikcevich, D.A., Finnegan, A., Chong, A.S., et al. Inhibition of interleukin-2 (IL-2)-stimulated tyrosine kinase activity by leflunomide. Agents Actions 1994; 41: C279-282.

26. Elder, R.T., Xu, X., Williams, J.W., et al. The immunosuppressive metabolite of leflunomide, A77 1726, affects murine T cells through two biochemical mechanisms. J Immunol 1997; 159: 22-27.

27. Manna, S.K. and B.B. Aggarwal. Immunosuppressive leflunomide metabolite (A77 1726) blocks TNF-dependent nuclear factor-kappa B activation and gene expression. J Immunol 1999; 162: 2095-2102.

28. Mladenovic, V., Domljan, Z., Rozman, B, et al. Safety and effectiveness of leflunomide in the treatment of patients with active rheumatoid arthritis: results of a randomized, placebo-controlled phase II study. Arthritis Rheum 1995; 38: 1595-1603.

29. Strand, V., Cohen, S., Schiff, M., et al. Treatment of rheumatoid arthritis with leflunomide compared to placebo or methotrexate. Arch Intern Med 1999; 159: 2542-2550.

30. Hossain, M.M., and D.M. Margolis. Inhibition of HIV replication by A77 1726, the active metabolite of leflunomide, in combination with pyrimidine nucleoside reverse transcriptase inhibitors. J Acquir Immune Defic Syndr 2001; 28: 199-201.

31. Schläpfer, E., Fischer, M., Ott, P., et al. Anti-HIV-1 activity of leflunomide: a comparison with mycophenolic acid and hydroxyurea. AIDS 2003; 17: 1613-1620.

32. Waldman, W.J., Knight, D.A., Lurain, N.S., et al. Novel mechanism of inhibition of cytomegalovirus by the experimental immunosuppressive agent leflunomide. Transplantation 1999; 68: 814-825.

33. Knight, D.A., Hejmanowski, A.Q., Dierksheide, J.E., et al. Inhibition of herpes simplex virus type 1 by the experimental immunosuppressive agent leflunomide. Transplantation 2001; 71: 170-174.

34. Smolen, J.S., Kalden, J.R., Scott, D.L., et al. Efficacy and safety of leflunomide compared with placebo and sulphasalazine in active rheumatoid arthritis: a double-blind, randomized, multicentre trial. European Leflunomide Study Group. Lancet 1999; 353: 259-266.

35. Maddison, P., Kiely, P., Kirkham, B., et al. Leflunomide in rheumatoid arthritis: recommendations through a process of consensus. Rheumatology 2005; 44: 280-286.

**11.0 Appendices**

11.1 Appendix A - Medications that are metabolized by CYP 2C9 and contraindicated during treatment period

## Oral hypoglycemic agents

Tolbutamide

Glipizide

Glyburide

## Angiotensin II blockers

Losartan

Irbesartan

## Other

Amitriptyline

Celecoxib

Fluoxetine

Fluvastatin

Phenytoin

Rosiglitazone

Tamoxifen

Torsemide

Warfarin

Note: NSAIDs (eg. diclofenac, ibuprofen, meloxicam, naproxen, piroxicam, suprofen) although metabolized by CYP 2C9, have been used safely in clinical trials in combination with leflunomide (28, 29, 34). Additionally a multidisciplinary panel of experts in rheumatoid arthritis has agreed that NSAIDs can be used safely in combination with leflunomide (35). Therefore, the use of NSAIDs, at FDA-approved doses, is allowed during this study.

**11.2 Appendix B – Medications that Have Interactions with Cholestyramine**

amiodarone

anisindione

aspirin

bezafibrate

carbamazepine

cephalexin

cerivastatin

chloroquine

chlorothiazide

cholecalciferol

clopamide

cyclosporine

diclofenac

dicumarol

digitoxin

digoxin

doxepin

doxercalciferol

entacapone

estrogens

fenofibrate

fluvastatin

furosemide

glipizide

hydrochlorothiazide

hydrocortisone

iopanoic acid

iron

levothyroxine

liothyronine

lorazepam

meloxicam

methacycline

methotrexate

metronidazole

mycophenolate mofetil

niacin

penicillin G

phenobarbital

phenprocoumon

phenylbutazone

phenytoin

piroxicam

pravastatin

progestins

raloxifene

sulindac

tenoxicam

tetracycline

thyroglobulin

troglitazone

tyropanoate sodium

ursodiol

valproic acid

warfarin

**11.3 Appendix C** **- CDC 1993 Revised Classification System for HIV Infection and Expanded Surveillance Case Definition for AIDS Among Adolescents and Adults**

**Category A**

Category A consists of one or more of the conditions listed below in an adolescent or adult (greater than or equal to 13 years) with documented HIV infection. Conditions listed in Categories B and C must not have occurred.

- Asymptomatic HIV infection
- Persistent generalized lymphadenopathy
- Acute (primary) HIV infection with accompanying illness or history of acute HIV infection Category B

**Category B**

Category B consists of symptomatic conditions in an HIV-infected adolescent or adult that are not included among conditions listed in clinical Category C and that meet at least one of the following criteria:

- the conditions are attributed to HIV infection or are indicative of a defect in cell-mediated immunity; or
- the conditions are considered by physicians to have a clinical course or to require management that is complicated by HIV infection.
- Examples of conditions in clinical Category B include, but are not limited to:
- Bacillary angiomatosis
- Candidiasis, oropharyngeal (thrush)
- Candidiasis, vulvovaginal; persistent, frequent, or poorly responsive to therapy
- Cervical dysplasia (moderate or severe)/cervical carcinoma in situ
- Constitutional symptoms, such as fever (38.5 C) or diarrhea lasting greater than 1 month
- Hairy leukoplakia, oral
- Herpes zoster (shingles), involving at least two distinct episodes or more than one dermatome
- Idiopathic thrombocytopenic purpura
- Listeriosis
- Pelvic inflammatory disease, particularly if complicated by tubo-ovarian abscess
- Peripheral neuropathy
- For classification purposes, Category B conditions take precedence over those in Category A. For example, someone previously treated for oral or persistent vaginal candidiasis (and who has not developed a Category C disease) but who is now asymptomatic should be classified in clinical Category B.

**Category C**

Category C includes the clinical conditions listed in the AIDS surveillance case definition. For classification purposes, once a Category C condition has occurred, the person will remain in Category C.

- Candidiasis of bronchi, trachea, or lungs
- Candidiasis, esophageal
- Cervical cancer, invasive *
- Coccidioidomycosis, disseminated or extrapulmonary
- Cryptococcosis, extrapulmonary
- Cryptosporidiosis, chronic intestinal (greater than 1 month's duration)
- Cytomegalovirus disease (other than liver, spleen, or nodes)
- Cytomegalovirus retinitis (with loss of vision)
- Encephalopathy, HIV-related
- Herpes simplex: chronic ulcer(s) (greater than 1 month's duration); or bronchitis, pneumonitis, or esophagitis
- Histoplasmosis, disseminated or extrapulmonary
- Isosporiasis, chronic intestinal (greater than 1 month's duration)
- Kaposi's sarcoma
- Lymphoma, Burkitt's (or equivalent term)
- Lymphoma, immunoblastic (or equivalent term)
- Lymphoma, primary, of brain
- Mycobacterium avium complex or M. kansasii, disseminated or extrapulmonary
- Mycobacterium tuberculosis, any site (pulmonary or extrapulmonary)
- Mycobacterium, other species or unidentified species, disseminated or extrapulmonary
- Pneumocystis carinii pneumonia
- Pneumonia, recurrent
- Progressive multifocal leukoencephalopathy
- Salmonella septicemia, recurrent
- Toxoplasmosis of brain
- Wasting syndrome due to HIV

**11.4 Appendix D –** **DAIDS Toxicity Table**

Estimating severity grade

For abnormalities NOT found elsewhere on the Toxicity Table use the scale below to estimate grade of toxicity:

GRADE 1 Transient or mild discomfort; no limitation in activity; no medical intervention/therapy required.

GRADE 2 Mild to moderate limitation in activity - some assistance may be needed;

no or minimal medical intervention/therapy required.

GRADE 3 Marked limitation in activity, some assistance usually required; medical intervention/therapy required, hospitalization possible.

GRADE 4 Extreme limitation in activity, significant assistance required; significant medical intervention/therapy required, hospitalization or hospice care.

| PARAMETER | **GRADE 1** | **GRADE 2** | **GRADE 3** | **GRADE 4** |
| --- | --- | --- | --- | --- |
| **HEMATOLOGY** |  | | | |
| Hemoglobin | 8.0 - 9.4 g/dL | 7.0 - 7.9 g/dL | 6.5 - 6.9 g/dL OR | <6.5 g/dL |
| **Absolute Neutrophil Count** | 1000 -1320/mm^3^ | 750 - 999/mm^3^ | 500 - 749/mm^3^ | <500/mm^3^ |
| **Platelets** | 75,000 - 99,000/mm^3^ | 50,000 - 74,999/mm^3^ | 20,000 - 49,999/mm^3^ | <20,000/mm^3^ |
| **Prothrombin Time (PT)** | >14.7 – 18.38 sec  (>1.0 – 1.25X ULN) | >18.38 – 22.05 sec  (>1.25 - 1.5 X ULN) | >22.05 – 44.1 sec  (>1.5 - 3.0 X ULN) | > 44.1 sec  (>3.0 X ULN) |
| **PTT** | >34.5 – 57.27 sec  (>1.0 - 1.66 X ULN) | > 57.27 – 80.39 sec  (>1.66 - 2.33 X ULN) | >80.39 – 103.5 sec  (>2.33 - 3.0 X ULN) | >103.5 sec  (>3.0 X ULN) |
| **Methemoglobin** | 5.0 - 10.0% | 10.1 - 15.0% | 15.1 - 20.0% | >20% |

| **PARAMETER** | **GRADE 1** | **GRADE 2** | **GRADE 3** | **GRADE 4** |
| --- | --- | --- | --- | --- |
| CHEMISTRIES |  | | | |
| SODIUM |  | | | |
| **Hyponatremia** | 130 - 134 meq/L | 123 - 129 meq/L | 116 - 122 meq/L | <116 meq/L |
| **Hypernatremia** | 146 - 150 meq/L | 151 - 157 meq/L | 158 - 165 meq/L | >165 meq/L |
| **POTASSIUM** |  | | | |
| **Hyperkalemia** | 5.6 - 6.0 meq/L | 6.1 - 6.5 meq/L | 6.6 - 7.0 meq/L | >7.0 meq/L |
| **Hypokalemia** | 3.0 - 3.2 meq/L | 2.5 - 2.9 meq/L | 2.0 - 2.4 meq/L | <2.0 meq/L |
| **PHOSPHATE** |  | | | |
| **Hypophospha-temia** | 2.0 - 2.2 mg/dL | 1.5 - 1.9 mg/dL | 1.0 - 1.4 mg/dL | <1.0 mg/dL |
| **CALCIUM -** | **Modified for NIH Clinical Center lab values** | | | |
| **Hypocalcemia** | 1.95 – 2.04 mmol/L | 1.75 – 1.925 mmol/L | 1.525 – 1.725 mmol/L | <1.525 mmol/L |
| **Hypercalcemia** | 2.65 – 2.875 mmol/L | 2.9 – 3.125 mmol/L | 3.15 – 3.375 mmol/L | >3.375 mg/dL |
| **MAGNESIUM** | **Modified for NIH Clinical Center lab values** | | | |
| **Hypomagne-semia** | 0.6 – 0.7 mmol/L | 0.45 – 0.55 mmol/L | 0.3 – 0.4 mmol/L | <0.3 meq/L |
| **BILIRUBIN** |  | | | |
| **Hyperbilirubin-emia** | >1.0 – 1.5 mg/dL  (>1.0 - 1.5 x ULN) | >1.5 – 2.5 mg/dL  (>1.5 - 2.5 x ULN) | >2.5 – 5 mg/dL  (>2.5 - 5 x ULN) | >5 mg/dL  (>5 x ULN) |
| **GLUCOSE** |  | | | |
| **Hypoglycemia** | 55 - 64 mg/dL | 40 - 54 mg/dL | 30 - 39 mg/dL /L | <30 mg/dL |
| **Hyperglycemia (nonfasting and no prior diabetes)** | 116 - 160 mg/dL | 161 - 250 mg/dL | 251 - 500 mg/dL | >500 mg/dL |
| **TRIGLYCE-RIDES** | --------------------- | 400 - 750 mg/dL | 751 - 1200 mg/dL | >1200 mg/dL |
| **CREATININE** | >1.4 – 2.1 mg/dL  (>1.0 - 1.5 x ULN) | >2.1 – 4.2 mg/dL  (>1.5 - 3.0 x ULN) | >4.2 – 8.4 mg/dL  (>3.0 - 6.0 x ULN) | >8.4  (>6.0 x ULN) |
| **URIC ACID** |  | | | |
| **Hyperuricemia** | 7.5 - 10.0 mg/dL | 10.1 - 12.0 mg/dL | 12.1 - 15.0 mg/dL | >15.0 mg/dL |

| **PARAMETER** | **GRADE 1** | **GRADE 2** | **GRADE 3** | **GRADE 4** |
| --- | --- | --- | --- | --- |
| CHEMISTRIES(continued) |  |  |  |  |
| **AST (SGOT)** | 43 – 85 u/L  (1.25 - 2.5 x ULN) | >85 – 170 u/L  (>2.5 - 5.0 x ULN) | >170 – 340 u/L  (> 5.0 - 10.0 x ULN) | >340 u/L  (>10.0 x ULN) |
| **ALT (SGPT)** | 51 – 103 u/L  (1.25 - 2.5 x ULN) | >103 – 205 u/L  (>2.5 - 5.0 x ULN) | >205 – 410 u/L  (> 5.0 - 10.0 x ULN) | >410 u/L  (>10.0 x ULN) |
| **GGT (Male)** | 65 – 130 u/L  (1.25 - 2.5 x ULN) | >130 – 260 u/L  (>2.5 - 5.0 x ULN) | >260 – 520 u/L  (> 5.0 - 10.0 x ULN) | >520 u/L  (>10.0 x ULN) |
| **GGT (Female)** | 47.5 – 95 u/L  (1.25 - 2.5 x ULN) | >95 – 190 u/L  (>2.5 - 5.0 x ULN) | >190 – 380 u/L  (> 5.0 - 10.0 x ULN) | >380  (>10.0 x ULN) |
| **Alkaline Phosphatase** | 145 – 290 u/L  (1.25 - 2.5 x ULN) | >290 – 580 u/L  (>2.5 - 5.0 x ULN) | >580 – 1160 u/L  (> 5.0 - 10.0 x ULN) | >1160 u/L  (>10.0 x ULN) |
| **CPK**  **Female**  **Male** | >1.25-2.5 x ULN  >315 – 630 U/L  >483-965 U/L | >2.5-5 x ULN  > 630 – 1260 U/L  > 965- 1930 U/L | >5-10 x ULN  >1260-2520 U/L  > 1930- 3860 U/L | >10 x ULN  >2520 U/L  > 3860 U/L |
| **PANCREATIC ENZYMES** |  | | | |
| **Amylase** | >143 – 214.5 u/L  (>1.0 - 1.5 x ULN) | >214.5 – 286 u/L  (>1.5 - 2.0 x ULN) | >286 – 715 u/L  (>2.0 - 5.0 x ULN) | >715 u/L  (>5.0 x ULN) |
| **Pancreatic amylase** | >54 – 81 u/L  (>1.0 - 1.5 x ULN) | >81 – 108 u/L  (>1.5 - 2.0 x ULN) | >108 – 270 u/L  (>2.0 - 5.0 x ULN) | >270 u/L  (>5.0 x ULN) |
| **Lipase** | >58 – 87 u/L  (>1.0 - 1.5 x ULN) | >87 – 116 u/L  (>1.5 - 2.0 x ULN) | >116 – 290 u/L  (>2.0 - 5.0 x ULN) | >290 u/L  (>5.0 x ULN) |

| **CARDIO-VASCULAR** | **GRADE 1** | **GRADE 2** | **GRADE 3** | **GRADE 4** |
| --- | --- | --- | --- | --- |
| **CARDIAC ARRHYTHMIA** | ------------------ | Asymptomatic; transient dysrhythmia, no Rx req | Recurrent/persistent dysrhythmia; symptomatic Rx req | Unstable dysrhythmia, hospitalization and Rx req |
| **HYPER-TENSION** | Transient, increase >20 mmHg; no Rx | Recurrent; chronic increase >20 mmHg, Rx required | Acute Rx req; outpatient hospitalization possible | Hospitalization required |
| **HYPO-TENSION** | Transient orthostatic hypotension, no Rx | Symptoms correctable with oral fluid Rx | IV fluid required, no hospitalization required | Hospitalization required |
| **PERICARDITIS** | Minimal effusion | Mild/mod asymptomatic effusion, no Rx | Symptomatic effusion, pain, EKG changes | Tamponade **OR** pericardiocen-tesis **OR** surgery required |
| **HEMOR-RHAGE, BLOOD LOSS** | --------------------- | Mildly symptomatic, no Rx required | Gross blood loss **OR** 1-2 units transfused | Massive blood loss **OR** >2 units transfused |

| **GASTRO-INTESTINAL** | **GRADE 1** | **GRADE 2** | **GRADE 3** | **GRADE 4** |
| --- | --- | --- | --- | --- |
| **NAUSEA** | Mild **OR** transient; reasonable intake maintained | Mod discomfort **OR** intake decreased for <3 days | Severe discomfort **OR** minimal intake for >3 days | Hospitalization required |
| **VOMITING** | Mild **OR** transient; 2-3 episodes per day **OR** mild vomiting lasting <1 week | Mod **OR** persistent; 4-5 episodes per day **OR** vomiting lasting >1 week | Severe vomiting of all food/fluids in 24 hrs **OR** orthostatic hypotension **OR** IV Rx required | Hypotensive shock **OR** hospitalization for IV Rx required |
| **DIARRHEA** | Mild **OR** transient; 3-4 loose stools per day **OR** mild diarrhea lasting <1 week | Mod **OR** persistent; 5-7 loose stools per day **OR** diarrhea lasting >1 week | Bloody diarrhea **OR** orthostatic hypotension **OR** >7 loose stools/day **OR** IV Rx required | Hypotensive shock **OR** hospitalization required |
| **ORAL DISCOMFORT/**  **DSYPHAGIA** | Mild discomfort, no difficulty swallowing | Difficulty swallowing but able to eat and drink | Unable to swallow solids | Unable to drink fluids; IV fluids required |
| **CONSTIPATION** | Mild | Moderate | Severe | Distention with vomiting |

| **NEUROLOGIC** | **GRADE 1** | **GRADE 2** | **GRADE 3** | **GRADE 4** |
| --- | --- | --- | --- | --- |
| **NEURO-CEREBELLAR** | Slight incoordination **OR** dysdiadochokin-esia | Intention tremor **OR** dysmetria **OR** slurred speech **OR** nystagmus | Locomotor ataxia requiring assistance to walk or arm incoordination interfering with ADLs | Unable to stand |
| **NEURO-PSYCH/MOOD** | --------------------- | --------------------- | Severe mood changes requiring medical intervention | Acute psychosis requiring hospitalization |
| PERIPHERAL NEUROPATHY | Mild discomfort; no treatment required | Moderate discomfort; non-narcotic analgesia required | Severe discomfort; **OR** narcotic analgesia required with symptomatic relief | Incapacitating discomfort; **OR** not responsive to narcotic analgesia |

| **NEUROLOGIC (continued)** | **GRADE 1** | **GRADE 2** | **GRADE 3** | **GRADE 4** |
| --- | --- | --- | --- | --- |
| NEURO-MUSCULAR PARESIS | Subjective weakness; no objective symptoms/signs | Mild objective signs, symptoms, no decrease in function | Objective weakness; function limited | Paralysis |
| NEURO-MOTOR | Decrease in reflexes **OR** patients with chronic stable abnormality of reflex or use of muscle | Absence of a previously present reflex | Absence of 2-3 previously present reflexes | Absence of >3 previously present reflexes |
| NEURO-SENSORY | Decrease in sensation (pinprick, vibratory or hot/cold) **OR** patients with chronic **STABLE** abnormality of sensation | Absence of a previously present sensory finding (one dermatome) | Absence of 2-3 previously present sensory dermatomes | Absence of >3 previously present sensory dermatomes |

| **RESPIRATORY** | **GRADE 1** | **GRADE 2** | **GRADE 3** | **GRADE 4** |
| --- | --- | --- | --- | --- |
| BRONCHO-SPASM, Acute | Transient; no Rx; FEV1 <80% - 70% (or peak flow) | Rx required; normalizes with bronchodilator; FEV1 50% - <70% (or peak flow) | No normalization with bronchodilator; FEV1 25% - <50% (or peak flow), retractions | Cyanosis; FEV1 <25% (or peak flow) **OR** intubated |
| DYSPNEA | Dyspnea on exertion | Dyspnea with normal activity | Dyspnea at rest | Dyspnea requiring O_2_ therapy |

| **URINALYSIS** | **GRADE 1** | **GRADE 2** | **GRADE 3** | **GRADE 4** |
| --- | --- | --- | --- | --- |
| PROTEINURIA |  |  |  |  |
| Spot urine | 1+ | 2 - 3+ | 4+ | Nephrotic syndrome |
| 24 hour urine | 200 mg-1 g loss/day **OR** <0.3% **OR** <3 g/l | > 1 - 2 g loss /day **OR** 0.3 - 1.0% **OR** 3-10g/l | > 2 - 3.5 g loss /day **OR** >1.0% **OR** >10 g/l | Nephrotic syndrome **OR** >3.5 g loss/day |
| GROSS HEMATURIA | Microscopic only | Gross, no clots | Gross plus clots | Obstructive **OR** transfusion req |

| **MISCEL-LANEOUS** | **GRADE 1** | **GRADE 2** | **GRADE 3** | **GRADE 4** |
| --- | --- | --- | --- | --- |
| **FEVER (oral, >12 hours)** | 37.7 - 38.5^0^C | 38.6 - 39.5^0^C | 39.6 - 40.5^0^C | >40.5^0^C for ≥ 12 continuous hours |
| **HEADACHE** | Mild; no Rx required | Moderate **OR** non-narcotic analgesia Rx | Severe **OR** responds to initial narcotic Rx | Intractable **OR** requiring repeated narcotic Rx |
| **ALLERGIC REACTION** | Pruritus without rash | Localized urticaria | Generalized urticaria angioedema | Anaphylaxis |
| **CUTANEOUS/RASH/**  **DERMATITIS** | Erythema, pruritus | Diffuse maculopapular rash **OR** dry desquamation | Vesiculation **OR** moist desquamation **OR** ulceration | **ANY ONE**: mucous membrane involvement, suspected Stevens-Johnson (TEN), erythema multiforme, necrosis req surgery, exfoliative dermatitis |
| **LOCAL REACTION (2% parenteral Rx - not vaccination or skin test)** | Erythema | Induration <10 mm **OR** inflammation **OR** phlebitis | Induration >10 mm **OR** ulceration | Necrosis of skin |
| **FATIGUE** | Normal activity reduced <25% | Normal activity reduced 25-50% | Normal activity reduced >50%; cannot work | Unable to care for self |
| **WEIGHT LOSS** | No criteria | 5-14% weight loss from baseline | >14-19% weight loss from baseline | ≥20% weight loss from baseline |
| **WEIGHT GAIN** | No criteria | 5-14% weight gain from baseline | >14-19% weight gain from baseline | ≥20% weight gain from baseline |
| **EDEMA** | Edema present, does not require therapy | Edema present, requires therapy | Edema unresponsive to therapy and/or requires discontinuation of investigational drug | Anasarca (severe generalized edema) |
| **ABDOMINAL PAIN** | Mild; no treatment required | Moderate; non-narcotic analgesia required | Severe; requires narcotics | Intractable or requires repeated narcotics |
| **HAIR LOSS** | Excessive hair on pillow on rising or mild hair loss from other regions | Hair can be easily pulled from scalp or moderate hair loss from other regions | Total alopecia or alopecia universalis | There is no Grade IV |
| **ANGIOEDEMA** | None | Swelling not involving airway resolves with antihistamine | Swelling of lips or tongue requiring antihistamines | Swelling of lips or tongue requiring epinephrine |
| **PULMONARY: RADIOGRAPHIC CHANGES** | Radiographic changes asymptomatic | Radiographic changes requiring treatment | Radiographic changes requiring oxygen | Radiographic changes requiring ventilation |
| **CARDIAC ISCHEMIA** | Non specific T wave flattening or changes | Asymptomatic ST T wave changes suggesting ischemia | Angina without infarction | Myocardial infarction |
| **AV HEART BLOCK** | Asymptomatic not requiring treatment | Symptomatic but not requiring treatment | Symptomatic and requiring treatment | Life threatening (with CHF, hypotension, syncope, shock) |
| **NODAL ARRHYTHMIA** | Asymptomatic not requiring treatment | Symptomatic but not requiring treatment | Symptomatic and requiring treatment | Life threatening (with CHF, hypotension, syncope, shock) |
| **SINUS BRADYCARDIA** | Asymptomatic not requiring treatment | Symptomatic but not requiring treatment | Symptomatic and requiring treatment | Life threatening (with CHF, hypotension, syncope, shock) |
| **SINUS TACHYCARDIA** | Asymptomatic not requiring treatment | Symptomatic but not requiring treatment | Symptomatic and requiring treatment | Life threatening (with CHF, hypotension, syncope, shock) |
| **SUPRAVENTRICULAR ARRHYTHMIA** | Asymptomatic not requiring treatment | Symptomatic but not requiring treatment | Symptomatic and requiring treatment | Life threatening (with CHF, hypotension, syncope, shock) |
| **VENTRICULAR ARRHYTHMIA** | Asymptomatic not requiring treatment | Symptomatic but not requiring treatment | Symptomatic and requiring treatment | Life threatening (with CHF, hypotension, syncope, shock) |
