# Supplementary material for: The Effect of Leflunomide on Cycling and Activation of T-Cells in HIV-1-Infected Participants
Source: PLoS One. 2010 Aug 3;5(8):e11937. doi: 10.1371/journal.pone.0011937 (PMC2914784; doi:10.1371/journal.pone.0011937)
Supplement: IRB Approval S1 — (0.06 MB PDF) [file pone.0011937.s002.pdf]

DEPARTMENT OF HEALTH AND HUMAN SERVICES
National Institutes of Health
National Institute of Allergy
and Infectious Diseases

DATE: 11/23/2004

TO: Sarah Wynne, M.D.
PRINCIPAL INVESTIGATOR

FROM: Chair, NIAID-IRB

APPROVAL LETTER

PROTOCOL NUMBER: 224
PROTOCOL TITLE: A Double-Blind, Randomized, Placebo-Controlled Study to Evaluate the Effect of Leflunomide on HIV-1 Associated Immune Proliferation in Vivo
MEETING DATE: 7/12/2004
EXPIRATION DATE: unassigned
ADULT RISK/BENEFIT CATEGORY: The research involves more than a minor increase over minimal risk to subjects (45 CFR 46.102(h)(i)). / The research involves no prospect of direct benefit to individual subjects but is likely to yield generalizable knowledge about the subject's disorder or condition (45 CFR 46.102(h)(i)).
CHILD RISK CATEGORY: Not Eligible
REQUEST: Initial Review
The purpose of this protocol is to evaluate the effect of the immunomodulatory agent, leflunomide, on CD4+ T cell proliferation in HIV infected adults.

All contingencies, if any, have been met and study activities may proceed. According to Federal Regulation (45CFR46), a continuing review of research shall be conducted at intervals appropriate to the degree of risk, but not less than once per year. The Institutional Review Board (IRB) office recommends submission of continuing review requests 6 weeks prior to the expiration date.
Changes in research activities during the approved IRB period shall not be initiated without prior IRB review and approval, except when necessary to eliminate apparent immediate hazards to the subject. Such amendments must be approved by the IRB prior to implementation.

Additionally, investigators must report to the IRB adverse events in accordance with the procedures outlined in the protocol.

For further guidance, current forms and instructions, please view the IRB web page at http://intramural.niaid.nih.gov/ocd/IRBweb/, or call the Human Subject Protections office at (301) 435-9273.

UPDATED CONSENT/ASSENT DISK(S) INCLUDED: _X_ YES ___ NO

[signature] 12·3·04
Peter Mannon, M.D. Date
Chair, NIAID-IRB

[signature] 12/20/04
H. Clifford Lane, M.D. Date
Clinical Director, NIAID

FOR OPS USE ONLY
Date: 1/6/05
Protocol #: 05-I-0065
Specialist: [signature]

NIAID IRB Tracking No. {3830 - 224}
