# Supplementary material for: The Effect of Leflunomide on Cycling and Activation of T-Cells in HIV-1-Infected Participants
Source: PLoS One. 2010 Aug 3;5(8):e11937. doi: 10.1371/journal.pone.0011937 (PMC2914784; doi:10.1371/journal.pone.0011937)
Supplement: CROI Abstract S1 — (0.06 MB PDF) [file pone.0011937.s003.pdf]

Session 722

A Study of Leflunomide to Target Immune Activation in HIV: Results of a Pilot Study

Sarah Read*, M De Grezia, E Ciccone, R DerSimonian, W Gao, C Rehm, A Pau, R Davey, C Lane, and I Sereti
NIAID, NIH, Bethesda, MD, US

Background: An increase in immune activation and turnover of CD4 T cells is considered to be a primary mechanism of HIV pathogenesis. Leflunomide is an immunomodulatory agent approved for use in rheumatoid arthritis that acts by decreasing turnover of activated lymphocytes via inhibition of dihydro-orotate dehydrogenase, the rate-limiting enzyme in the *de novo* synthesis of pyrimidines.

Methods: We performed a randomized, double-blind, controlled trial (ALETHIA) to evaluate the safety and effect of leflunomide on CD4 cell turnover in HIV-1-infected subjects. Subjects with CD4 counts ≥350 cells/μL who were not receiving ART were treated with either leflunomide 20 mg/day or placebo for 28 days. On day 28, the treatment arm was unblinded to allow patients who received leflunomide to undergo wash-out treatment with cholestyramine. Toxicity data, CD4 and CD8 T cell counts ,and viral load were followed. Ki67 expression, BRDU incorporation and activation markers on T cells were examined by flow cytometry. Sign and Wilcoxon rank-sum tests were used for comparisons. Final results are reported here.

Results: We randomized 12 subjects to receive leflunomide, and 6 subjects to receive placebo. There were no differences between groups in median age (39.5 vs 39.0 years), baseline CD4 count (637 vs 434 cells/μL), viral load (3.87 vs 4.31 $\log_{10}$ copies/mL), or expression of Ki67 on CD4 T cells (4.3 vs 5.6%). The median leflunomide level in the treated group was 21.5 mg/L. A significant decrease in Ki67 expression was seen in the leflunomide group after 28 days of treatment (–0.8%; $p = 0.02$) and was not seen in the placebo group (–0.05%; $p = 1$). The between-group comparison was not significant for change in CD4 expression of Ki67 ($p = 0.55$), however, there was a significant difference between the 2 groups in change in BRDU incorporation in CD4 (–0.0785% vs 0.085%; $p = 0.03$). Additionally, the percentage of CD8 cells expressing CD38 and HLA-DR decreased in the leflunomide group (–5%; $p = 0.02$). Although HIV viral load decreased at day 15 in the leflunomide group (-0.152 $\log_{10}$ copies/mL; $p = 0.02$), a significant decrease was not seen at day 29 (–0.124 $\log_{10}$ copies/mL; $p = 0.34$). There were no grade 3 or 4 adverse events seen in the leflunomide group.

Conclusions: Leflunomide given for 28 days was safe and well tolerated in HIV-infected subjects who were not receiving ART. Additionally, it was effective in decreasing the turnover of $CD4^+$ T cells.
