# Supplementary material for: The Effect of Leflunomide on Cycling and Activation of T-Cells in HIV-1-Infected Participants
Source: PLoS One. 2010 Aug 3;5(8):e11937. doi: 10.1371/journal.pone.0011937 (PMC2914784; doi:10.1371/journal.pone.0011937)
Supplement: Consent S1 — (0.12 MB DOC) [file pone.0011937.s004.doc]

**INTRODUCTION**

We invite you to take part in a research study at the National Institutes of Health (NIH).

First, we want you to know that:

Taking part in NIH research is entirely voluntary.

You may choose not to take part, or you may withdraw from the study at any time. In either case, you will not lose any benefits to which you are otherwise entitled. However, to receive care at the NIH, you must be taking part in a study or be under evaluation for study participation.

You may receive no benefit from taking part. The research may give us knowledge that may help people in the future.

Second, some people have personal, religious or ethical beliefs that may limit the kinds of medical or research treatments they would want to receive (such as blood transfusions). If you have such beliefs, please discuss them with your NIH doctors or research team before you agree to the study.

Now we will describe this research study. Before you decide to take part, please take as much time as you need to ask any questions and discuss this study with anyone at NIH, or with family, friends or your personal physician or other health professional.

**PURPOSE OF THE STUDY**

The purpose of this study is to evaluate the effect of leflunomide, an immunomodulatory drug (that can fine tune the immune system), on the life cycle of CD4+ T cells in persons infected with HIV. Leflunomide (also called Arava®) is a drug which has been approved by the Food and Drug Administration (FDA) for the treatment of rheumatoid arthritis. It works by blocking the cell division of activated T cells. In HIV infection, the HIV virus causes increased activation of T cells. Activated CD4+ T cells become more easily infected by HIV and die. Also, this activation and division of T cells possibly can cause death of T cells even if they are not infected with the virus. T cells are necessary for the body to fight infections and cancer. This loss (death) of T cells is what causes a weakened immune system in people infected with HIV so that they are more likely to get infections or cancers.

If an immunomodulatory drug such as leflunomide could be used to block the division of activated T cells, it could decrease the number of cells that die because of continuous division, and also decrease the cells in which HIV can reproduce leading to a lower level of HIV virus in the body. Leflunomide has not been studied before in people infected with HIV so it is not known exactly what effect it will have on the immune system. Blocking the division of activated T cells in patients with HIV may lead to more suppression of the immune system.

In this study, we want to measure the cell division and death or “turnover” of CD4+ T cells in HIV infected persons who are treated with leflunomide as compared to those who are taking placebo (an inactive pill that resembles leflunomide). We will also be looking at things such as CD4+ T cell counts and viral loads that may be changed by leflunomide. Our goal is to enroll 18 subjects.

**STUDY ENTRY CRITERIA**

You are eligible for this study because you are between the ages of 18 and 65, have a diagnosis of HIV, and have HIV viral levels greater than or equal to 1000 copies/mL when measured at the time of screening. You also have a CD4+ T cell count that is greater than or equal to 350 cells/mm^3^ and has never been less than 200 cells/mm^3^. You have a primary care physician who is taking care of your HIV infection. You agree to use two forms of approved contraception, and you are not planning on becoming pregnant or making a woman pregnant during the study period. **(Note: This applies to both men and women. Contraception is required during the 28 days of treatment for all participants. If you receive leflunomide you will also be required to use contraception until your leflunomide blood levels are undetectable (28 days or more following the treatment period.)**

For your safety, you are not eligible for this study if

- You are currently receiving antiretroviral therapy, have received it within 12 weeks of screening, or plan to start it within the study period
- You have previously received leflunomide or IL-2 at any time, you have received another immunomodulatory drug in the past 60 days (hydroxyurea, HIV vaccines, cyclosporine, mycophenolate mofetil, rapamycin, tacrolimus, sirolimus), or you have received corticosteroids (such as prednisone) by mouth or injection in the past 30 days
- You are unwilling or unable to stop taking drugs for diabetes, thyroid disease, cholesterol, or tuberculosis (*note: a complete list of medications that are not allowed will be discussed with you, and your medications will be reviewed with you as well*).
- You are currently using cholestyramine or know that you cannot tolerate it
- You have a history of familial hyperlipoproteinemia type III, IV or V
- You have had a bacterial infection in the past 4 weeks
- You have a history of an AIDS defining illness (as determined by the study investigator)
- You have hepatitis B or C or any other liver disease
- You have a history of biliary obstruction
- You are unwilling to stop drinking alcohol during the study period
- You have a history of high blood pressure that is not controlled on one medication
- You have abnormalities of your blood counts, liver tests, pancreas tests, kidney tests or blood clotting times
- You have a history of cancer and have not been free of disease for more than 5 years
- You have a serious medical or psychiatric condition that the study team feels will interfere with your ability to participate or would compromise your safety
- You are pregnant or breast-feeding
- You have a history of interstitial lung disease

**STUDY DESIGN**

This study requires participants to come to clinic at least 3 times and possibly up to 7 times for visits that typically take between 2 to 5 hours. The length of the study will be approximately 30 to 60 days. There will be approximately 18 patients enrolled on this study; approximately 12 will receive leflunomide and 6 will receive placebo.

If you fulfill the study entry criteria for this study, you will be randomized (by chance, like flipping a coin) to take either leflunomide 20 mg or placebo every day for 28 days. Your chances of taking leflunomide are 2 out of 3 or 67%, and your chances of taking placebo are 1 out of 3 or 33%. Neither you, your physician or any member of the study team will know whether you are receiving leflunomide or placebo up until day 28.

You will be asked to come in for clinic visits 3 times during the first 29 days of the study (day 1, 15 and 29). During the clinic visits, your history will be reviewed, particularly for any side effects (unpleasant symptoms) you may be experiencing. You will have a physical exam (a short exam on Day 1 and 15 and a more detailed exam on Day 29) and have blood drawn for safety laboratory testing as well as for research.

On Day 29, you and the study team will be informed whether you had been taking leflunomide or placebo. In other words, your treatment assignment will be “unblinded”. If you were receiving placebo, Day 29 will be your last study visit.

If you were receiving leflunomide, you will next take cholestyramine 8 grams three times a day for 11 days out of the next 14 days. The reason for giving you cholestyramine is to clear the leflunomide from your body. Without cholestyramine treatment, leflunomide will stay in your body for many months or even a year. With cholestyramine treatment, the amount of leflunomide in your body should be nearly undetectable at the end of 11 days. After you complete the cholestyramine treatment, you will return on Day 43 to have a leflunomide level checked to make sure only very little or no leflunomide remains in your body. If the level is low, you will end study on or around Day 64 (when the level is available). If this level remains elevated however, you will have to repeat the treatment with cholestyramine.

If you choose to withdraw from the study early or are asked by the study team to end the study early, the study team will learn whether you have been taking leflunomide or placebo at the time you withdraw from the study. You will have safety labs and pregnancy testing if applicable. If you were taking leflunomide, you will need to take cholestyramine 8 grams three times a day for 11 days to clear the leflunomide from your system. A leflunomide level will be drawn after you finish the cholestyramine to make sure only very little or no leflunomide remains in your body. If this level remains elevated, you will have to repeat the treatment with cholestyramine.

**During this study, strict contraceptive methods are required by all participants, whether male or female. This means two reliable methods of birth control (hormonal such as birth control pills, patches or injections, intrauterine device, barrier mechanical methods such as latex condoms or diaphragms).** **Contraception is required during the 28 days of treatment for all participants. If you receive leflunomide you will also be required to use contraception until your leflunomide blood levels are undetectable (28 days or more following the treatment period).**

**Stored Samples**

During your participation in this screening and on this study, blood will be collected by standard blood drawing techniques. Some blood samples will be used for this research study. We will also store some of your blood samples. These samples will be kept and used in future research to learn more about HIV, the immune system and/or other medical conditions.

Generally, the results from the research done with your stored samples will not be given to your private doctor and will not be put in your medical record. This is because the test results, unlike routine medical testing, may be experimental or preliminary. The relevance of these tests to your care may be unknown.

# Labeling of stored samples

Your stored samples will be labeled with a code (such as a number) that only the study team can link to you or an identifier (your name). Any identifying information about you will be kept confidential to the extent permitted by law.

# Future Studies

In the future, other investigators (at NIH or outside of NIH) may wish to study your stored samples for studies unrelated to the current protocol. When the study team shares your materials, they may share it with no identifying information or with a code. Some information about you, such as your gender, age, health history, or ethnicity may also be shared with other investigators. Any future research using your samples in studies unrelated to the current protocol will be reviewed by the investigator’s Institutional Review Board (IRB), a special committee that oversees medical research studies to protect the rights and welfare of human subject volunteers.

Your stored materials will be used only for research and will not be sold. The research done with your materials may be used to develop new products in the future but you will not receive payment for such products.

# RISKS AND/OR DISCOMFORTS

Once enrolled on this study, you will be required to come to the NIH for 3 visits in a 29 day period. If you are randomized to receive leflunomide, you will be required to come for at least two additional visits while receiving cholestyramine. These visits will involve a review of your medical history, a physical examination and blood draw for safety monitoring and research and may be time consuming and inconvenient.

The major risks of this study are those associated with leflunomide and cholestyramine.

# Potential Risks associated with Leflunomide:

Leflunomide has been associated with certain side effects. The most serious side effects are the following:

- Immunosuppression potential/Bone marrow suppression:
  - There have been rare reports of low red blood cell, white blood cell and platelet counts in patients receiving leflunomide alone. These events have been reported most frequently in patients who received treatment with other medication such as methotrexate in addition to leflunomide. In some cases, patients had a prior history of a significant abnormality related to these cells in the blood.
  - Rarely serious infections, which may be fatal, have been reported in patients receiving leflunomide. Most of the reports were in patients who were taking other therapy that suppressed the immune system and/or had other illnesses that may have made them more likely to get an infection.
  - The risk of malignancy (cancer), particularly some kinds of leukemias (cancer of the white blood cells) and lymphomas (cancers of the immune system), is increased with the use of some immunosuppression medications. There is a potential for immunosuppression with leflunomide. No apparent increase in the incidence of malignancies was reported in the clinical trials of leflunomide, but larger and longer-term studies would be needed to determine whether there is an increased risk of cancer with leflunomide.
- Liver Toxicity:
  - Rare cases of severe liver injury, including with fatal outcome, have been reported during treatment with leflunomide. Most cases of severe liver injury occur within 6 months of therapy and in a setting of multiple risk factors for damage to the liver (for example underlying liver disease or on treatment with other drugs which are toxic to the liver). In clinical trials, leflunomide treatment alone or in combination with methotrexate was associated with elevations of liver enzymes in a significant number of patients; these effects were generally reversible. Most liver enzyme elevations were mild and usually went away while continuing treatment. Marked elevations (which may indicate liver damage) occurred infrequently and reversed with lowering the dose of leflunomide or stopping it.
- Skin Reactions
  - Rare cases of Stevens-Johnson syndrome and toxic epidermal necrolysis have been reported in patients receiving leflunomide. These are very serious forms of allergic reactions that involve the skin. They cause intense reddening and blistering of the skin that will lead to peeling of the skin over large portions of the body. These reactions can be fatal.
- Fetal Death/Birth Defects
  - There are no studies evaluating leflunomide in pregnant women, however based on animal studies, leflunomide may increase the risk of fetal death or birth defects when used in pregnancy. When given to rats, leflunomide caused birth defects such as anophthalmia (absence of eyes), microophthalmia (unnaturally small size of eyes), and hydrocephalus (accumulation of fluid in the brain). It also caused decreased weight in the pregnant rats and increased rate of death of the rat fetuses as well as decreased weight of the surviving fetuses. In rabbits, leflunomide caused abnormal bone formation in fetuses. For these reasons, leflunomide is not to be used in pregnant women, and men and women receiving leflunomide should use strict birth control methods.
- Interstitial lung disease
  - Interstitial lung disease is disease that results in scarring of the lungs and can be fatal. Interstitial lung disease has been reported during treatment with leflunomide.and in some cases has been fatal. It may occur at any time during treatment and may present in different ways. New or worsening respiratory symptoms, such as cough or difficulty breathing, may be reason to stop treatment with leflunomide.

The most common side effects are the following:

- Diarrhea
- Nausea
- Rash
- Hair loss
- Headache

Other side effects have been reported in controlled studies of leflunomide in the treatment of rheumatoid arthritis and may or may not be related to leflunomide. These include the following:

- Allergic reaction (1-5%)
- Loss of strength or energy (3-6%)
- Flu syndrome (0-4%)
- Infection (0-4%)
- Injury/accident (5-7%)
- Pain (1-4%)
- Stomachache or upset stomach (5-10%)
- Back pain (5-8%)
- High blood pressure (9-10%)
- Chest pain (1-2%)
- Loss of appetite (3%)
- Mouth ulcers (3-5%)
- Vomiting (3-5%)
- Low potassium level (1-3%)
- Weight loss (2-4%)
- Joint pain or swelling (1-8%)
- Leg cramps (0-4%)
- Dizziness (4-7%)
- Numbness or tingling (2-4%)
- Bronchitis (5-8%)
- Increased cough (3-5%)
- Respiratory infection (15-27%)
- Throat infection (2-3%)
- Pneumonia (2-3%),
- Runny nose (2-5%)
- Sinus infection (1-5%)
- Eczema (a type of skin rash) (1-3%)
- Itching (4-6%)
- Dry skin (2-3%)
- Urinary tract infection (5%)

Since the licensing of leflunomide for public use, the following side effects have been reported rarely and may or may not be related to leflunomide:

- Body as a whole: serious infections that may be fatal
- Gastrointestinal: pancreatitis (inflammation of the pancreas)
- Hematologic: low white blood cell count, low neutrophil count, low platelet count
- Hypersensitivity: angioedema (swelling of skin and tissue under the skin)
- Liver: hepatitis (inflammation of the liver), jaundice, severe liver injury that may be fatal
- Respiratory: interstitial lung disease (lung disease that results in scarring of the lungs) that may be fatal
- Nervous system: peripheral neuropathy (numbness and tingling in the toes and feet)
- Skin and appendages: erythema multiforme, Stevens-Johnson syndrome, toxic epidermal necrolysis (these conditions are described on page 4 under Skin reactions).

**Potential Risks associated with Cholestyramine:**

The most common side effects caused by cholestyramine are gastrointestinal and include constipation, stomachache, nausea, gas, vomiting, diarrhea, heartburn, loss of appetite, and indigestion. Mild steatorrhea (fat in the stool) may also occur. Constipation is the most common complaint in cholestyramine users. Complete intestinal obstruction has been reported in a couple of patients.

Other side effects of cholestyramine include rare reports of abnormalities of the cells in the blood. Bleeding tendencies due to hypoprothrombinemia (low level of a clotting factor) have been reported as well as one case of abnormally large platelets.

A metabolic disorder called hyperchloremic acidosis (a disorder in which the kidneys are unable to remove acids from the blood) has been reported to occur in small children and older adults receiving treatment with a medication called spironolactone at the same time as receiving cholestyramine. Decreased thyroid function has been reported in a small number of patients who were already diagnosed with decreased thyroid function and were receiving thyroid hormone replacement. This effect may be due to a drug interaction between cholestyramine and thyroid hormone.

Cholestyramine may interfere with normal fat absorption and thus prevent absorption of fat soluble vitamins A, D, E, and K.

Cholestyramine does not fully dissolve and can be unpleasant to drink. Keeping cholestyramine in the mouth for a long time may lead to change in tooth color or damage or weakening of teeth.

There is one reported case of urinary tract stone associated with cholestyramine use.

Cholestyramine may interact with some drugs. There are additive effects with cholestyramine and HMG-CoA reductase inhibitors and nicotinic acid (medications used for lowering cholesterol). Cholestyramine may decrease absorption of several medications.

Although the safe use of cholestyramine during pregnancy has not been fully established, the risks involved with its use in pregnant patients appear to be minimal.

**Risks associated with blood drawing:**

Blood drawing from a vein may cause some discomfort, and occasionally some bruising or bleeding at the site. Rarely people may faint while having their blood drawn. Rarely there are infections of the skin that result from blood draws. Blood drawing will not exceed 450 milliliters (1 pint) over any six week period, which is within the safety guidelines for blood drawing practiced at the Clinical Center.

**Risks associated with stored specimens:**

The greatest risk is the unplanned release of information from your medical records. The chance that this information will be given to an unauthorized person without your permission is very small. Possible problems with the unplanned release of information include discrimination when applying for insurance or employment. Similar problems may occur if you disclose information yourself or agree to have your medical records released.

**BENEFITS AND COMPENSATION**

**Benefits:**

There are no clinical benefits to you by participating in this study. However, what we learn from this study may help us to improve treatment of people who are infected with HIV in the future.

**Compensation:**

Compensation in the amount of $640 will be provided for your participation in the entire study. This amount is based on the number of blood draws obtained and the number of study visits required as well as the inconvenience to you. If you do not complete the entire study, you will be compensated for blood draws obtained and study visits made based on the NIH guidelines for clinical research volunteer program.

**Costs to You for Participating in this Study:**

We will provide clinical and professional services, diagnostics, and lab work that are part of the study and not part of your regular care at no cost to you. Participants will receive partial reimbursement consistent with the NIAID travel reimbursement policy for any immediate costs associated with study-related expenses (eg. travel costs, lodging, etc.). It is possible that you may be required to assume financial responsibility for some expenses that are not covered under the policy.

# ALTERNATIVES TO PARTICIPATION

An alternative to participating in this study is simply to continue to be followed by your private physician for care related to HIV infection. There are also a number of experimental protocols using different combination of both licensed and investigational agents that are being investigated in many medical centers across the country, and you may be eligible for some of these studies.

# NEW FINDINGS

You will be told of any new information learned during the study that might cause you to change your mind about staying in the study. At the end of the study you will be told when the study results may be available and how to learn about them.

# CRITERIA FOR EARLY WITHDRAWAL FROM STUDY

You will be removed from the study if you become pregnant. You may also be removed if you develop cancer or a serious infection, a rise in your liver enzymes, a serious side effect or allergic reaction. In addition, you may have to leave the study if the study team feels that staying in the study is harmful to you, you do not comply with the study requirements, or the study is stopped.

# CONFIDENTIALITY

Your medical records will be kept confidential to the extent permitted by law. Your name or any other data that might identify you will not be used in any reports or publications resulting from this study. Because of your consent to participate, your medical records may be reviewed by the NIH staff, by the FDA, and by the Institutional Review Board of the NIH (the body responsible for ensuring that the study complies with the guidelines for the protection of human research subjects) with the understanding that these records will be used only in connection with carrying out obligations related to this clinical study. We will be routinely supplying information about you to your personal physician(s). You may change physicians whenever you like, but we need to have a physician with whom to communicate about you. We would not ask your permission for each of these contacts.

# ADDITIONAL INFORMATION

During the study, the NIH Clinical Center will provide only your study-related care, including evaluation of complications that may be study related. If you require medical care at other hospitals during the study, you or your insurance may be charged for this care. During the study, medical care and medications not directly related to the study must continue to be provided by your personal physician. Once your participation in this study is completed, you will not be eligible for

continuing care at the NIH. However, your doctors at the NIH will be glad to provide telephone consultation to your home doctor upon request throughout the course of your illness.

**OTHER PERTINENT INFORMATION**

**1. Confidentiality.** When results of an NIH research study are reported in medical journals or at scientific meetings, the people who take part are not named and identified. In most cases, the NIH will not release any information about your research involvement without your written permission. However, if you sign a release of information form, for example, for an insurance company, the NIH will give the insurance company information from your medical record. This information might affect (either favorably or unfavorably) the willingness of the insurance company to sell you insurance.

The Federal Privacy Act protects the confidentiality of your NIH medical records. However, you should know that the Act allows release of some information from your medical record without your permission, for example, if it is required by the Food and Drug Administration (FDA), members of Congress, law enforcement officials, or other authorized people.

**2. Policy Regarding Research-Related Injuries.** The Clinical Center will provide short-term medical care for any injury resulting from your participation in research here. In general, no long-term medical care or financial compensation for research-related injuries will be provided by the National Institutes of Health, the Clinical Center, or the Federal Government. However, you have the right to pursue legal remedy if you believe that your injury justifies such action.

**3. Payments.** The amount of payment to research volunteers is guided by the National Institutes of Health policies. In general, patients are not paid for taking part in research studies at the National Institutes of Health.

**4. Problems or Questions.** If you have any problems or questions about this study, or about your rights as a research participant, or about any research-related injury, contact the Principal Investigator Dr. Irini Sereti, the National Institute of Allergy and Infectious Diseases, 9000 Rockville Pike Building: 10 , Room 11B05 , Bethesda, MD 20892; Telephone: 301-496-5533 (Daytime phone number), or 301-496-1211 (24 hour contact number), or Mary DeGrezia, R.N., M.S., Research Study Coordinator, at 301-496-7040 (Daytime phone number).

You may also call the Clinical Center Patient Representative at 301-496-2626.

**5. Consent Document.** Please keep a copy of this document in case you want to read it again.

| **COMPLETE APPROPRIATE ITEM(S) BELOW:** | |  | |
| --- | --- | --- | --- |
| **A. Adult Patient’s Consent** | **B. Parent’s Permission for Minor Patient.** |  | |
| I have read the explanation about this study and have been given the opportunity to discuss it and to ask questions. I hereby consent to take part in this study. | I have read the explanation about this study and have been given the opportunity to discuss it and to ask questions. I hereby give permission for my child to take part in this study.  (Attach NIH 2514-2, Minor’s Assent, if applicable.) |  | |
|  |  |  | |
| Signature of Adult Patient/Legal Representative Date | Signature of Parent(s)/Guardian Date |  | |
|  |  |  | |
| **C. Child’s Verbal Assent (If Applicable)**  The information in the above consent was described to my child and my child agrees to participate in the study. | |  |  |
|  |  |  | |
| Signature of Parent(s)/Guardian Date |  |  | |
|  |  |  | |
| **THIS CONSENT DOCUMENT HAS BEEN APPROVED FOR USE  FROM JULY 12, 2006 THROUGH JULY 12, 2007.** | |  |  |
|  |  |  | |
| Signature of Investigator Date | Signature of Witness Date |  | |
